# Proton-enabled activation of peptide materials for biological bimodal memory

**DOI:** 10.1038/s41467-020-19750-5

**Published:** 2020-11-19

**Authors:** Min-Kyu Song, Seok Daniel Namgung, Daehwan Choi, Hyeohn Kim, Hongmin Seo, Misong Ju, Yoon Ho Lee, Taehoon Sung, Yoon-Sik Lee, Ki Tae Nam, Jang-Yeon Kwon

**Affiliations:** 1 School of Integrated Technology, Yonsei University, Incheon, 21983 Republic of Korea; 2 Department of Materials Science and Engineering, Seoul National University, Seoul, 08826 Republic of Korea; 3 School of Chemical and Biological Engineering, Nano Systems Institute, Seoul National University, Seoul, 08826 Republic of Korea

**Keywords:** Biopolymers, Electronic devices

## Abstract

The process of memory and learning in biological systems is multimodal, as several kinds of input signals cooperatively determine the weight of information transfer and storage. This study describes a peptide-based platform of materials and devices that can control the coupled conduction of protons and electrons and thus create distinct regions of synapse-like performance depending on the proton activity. We utilized tyrosine-rich peptide-based films and generalized our principles by demonstrating both memristor and synaptic devices. Interestingly, even memristive behavior can be controlled by both voltage and humidity inputs, learning and forgetting process in the device can be initiated and terminated by protons alone in peptide films. We believe that this work can help to understand the mechanism of biological memory and lay a foundation to realize a brain-like device based on ions and electrons.

## Abstract (web summary)

The structural programmability and functionality of peptide materials can be leverage for various next-generation devices such as non-volatile memories. The authors report a proton-coupled mechanism in tyrosine-rich peptides for realizing multimodal memory devices.

## Introduction

The cooperative and multimodal activation process, which includes the transfer of ions or small molecules, specific recognition by receptors and subsequent cascade reactions by at least two inputs, is one of the most important characteristics of biological memory. For example, in a neuronal synapse, many chemicals such as neurotransmitters (e.g., dopamine), calcium ions, and protons cooperate in the process of electrical signal transfer, which, interestingly, depends on the external factors determined by sensory input or the memory of the previous events^1,2^. However, there is a large gap in our understanding and in controllability between electron-based synthetic devices and ion-based synaptic operation. Despite the advantage of the ability to harness coupled behaviors of ions and electrons as inputs, there are few reports of the application of such concerted behaviors to brain-inspired devices.

The proton-coupled mechanism that we focus on is ubiquitous in biological systems, as represented by proton-coupled electron transfer (PCET)^3,4^. Moreover, the role of protons in the synapse as neurotransmitters and mediators in cooperation with other neurotransmitters has been investigated in the past^5,6^. Proton-mediated signaling, in conjunction with conventional neurotransmitters, has been found mostly in sensory neurons in conditioned learning. Transient changes in extracellular proton concentrations are important in the activation of further signaling^7,8^. In this regard, along with the dopamine-based reward mechanism, proton-based processes can be applied to novel algorithms or synaptic devices. Our approach is based on one of the lessons learned from recent biological discoveries^3–8^: that protons can facilitate metal redox and accumulate locally in electrically insulating peptide films.

The advantages of peptide materials are their structural programmability and versatile functionality, which can be controlled at a sequence level. Sequences can control folding, assembly, and other properties including inorganic material growth and electronic/ionic conductivities^9–12^. In this regard, peptides have been utilized even for nonbiomedical applications, including transistors, light-emitting devices, solar cells, batteries, and piezoelectric devices^13–16^. Previously, we identified the Tyr–Tyr–Ala–Cys–la-Tyr–Tyr (YYACAYY, Y7C) peptide, which can form a helix dimer and assemble spontaneously into a 2-D film^17^. We have investigated the proton conductivity in manganese oxide hybridized Y7C films^18^. Through the measurement of the Onsager coefficient to understand the coupling of electrons and protons, it became evident that tyrosine can be involved in PCET and metal ion redox even in the thick films^19^. Thus, we explored the possibility of using the Y7C peptide for proton-mediated memory and synaptic devices.

Here, we demonstrate a programmable approach to proton-regulated control based on tyrosine-rich peptide (TRP), providing a platform for designing and evaluating a bioinspired process of memory and learning algorithms. In this study, TRP is assembled into a film, and proton-dependent plasticity in conduction is utilized to activate memory and synaptic performance. We are further able to control the proton-controlled memory process and the timescale of learning and forgetting, which have never been realized with other materials.

## Results

### Film formation and electrochemical characterization

**Fig. 1:**
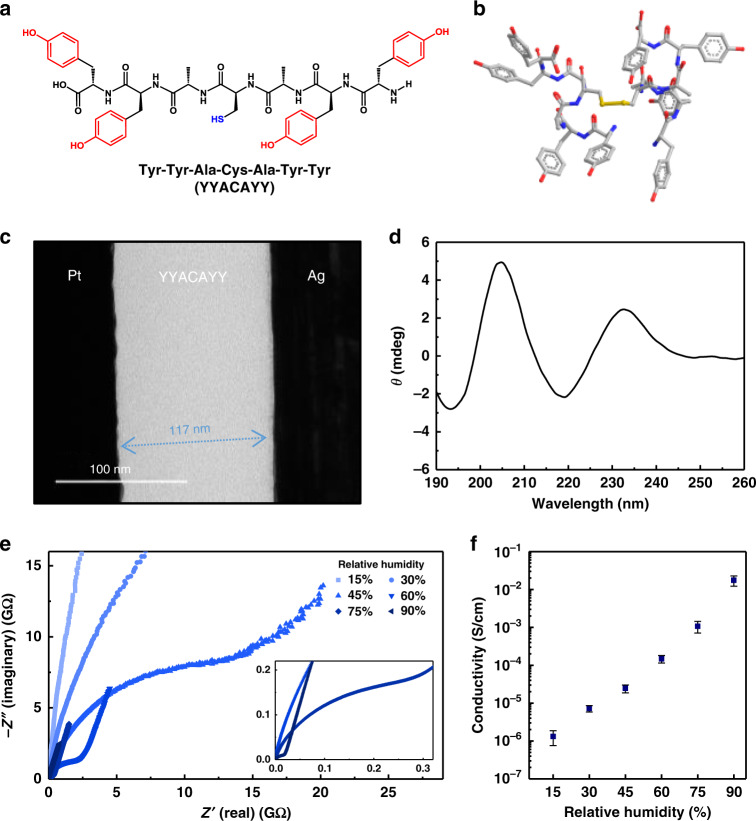
Film formation and proton conduction of the Y7C peptide. (**a**) Chemical structure of the Y7C peptide. The phenolic groups of tyrosine and thiol groups of cysteine are highlighted in red and blue, respectively. (**b**) Molecular structure of the dimer of Y7C revealed by NMR. Carbon, oxygen, and nitrogen are shown in gray, red, and purple, respectively. (**c**) Cross-sectional HRTEM image of the Y7C film between Pt and Ag electrodes. A uniform and homogeneous film with a thickness of 117 nm can be identified between the Pt bottom electrode and the Ag top electrode. (**d**) Circular dichroism (CD) analysis of the Y7C film. The solid CD spectrum is expressed in degrees of ellipticity (θ) without concentration terms. (**e**) Nyquist plots measured from the Y7C film in contact with two Au electrodes at different RH. The inset shows the magnified range at RH values of 60%, 75%, and 90%. (**f**) Conductivity of the Y7C film calculated from the Nyquist plot as a function of RH. Error bars represent the mean ± SD.

The Y7C peptide can exist as a dimer by disulfide bonding, exhibiting a helical structure, as confirmed by NMR (Fig. 1a, b). Because of the structural stability, this peptide can easily be assembled and deposited as a thick film by the spin-coating process (Fig. 1c). Circular dichroism analysis showed that the Y7C film had a helical conformation despite being made by spin-coating followed by a quick-drying process (Fig. 1d). Note that TRP exhibits different optical rotatory properties from typical helical peptides because of the absorbance of tyrosine groups^17,20,21^. The strong positive peak at 205 nm can be assigned to the amide π–π* transition and the π–π* transition of the phenolic side chain in an α-helical conformation at approximately 200 nm^20^. In addition, the positive peak at 232 nm found in the Y7C film corresponds to another π-π* transition of the phenolic side chain at 230 nm previously reported for Y7C peptide in aqueous solution with an α-helical conformation^17^.

The proton conduction property of the Y7C peptide was investigated using electrochemical impedance analysis (EIS). Ion-blocking gold electrodes were utilized to measure capacitive effects solely from protons. Alternating current (AC) was applied with frequencies ranging from 20 Hz to 10^6^ Hz to obtain complex impedance plots with real parts on the x-axis and imaginary parts on the y-axis, known as Nyquist plots (Fig. 1e). Proton conductivity was estimated by fitting the Nyquist plot to a typical resistor-capacitor (RC) circuit used for modeling proton exchange membranes (Supplementary Fig. 1 and Supplementary Note 1)^22–24^. The Y7C film showed an increase in conductivity as the relative humidity (RH) increased (Fig. 1f and Supplementary Fig. 2). At 90% RH, the proton conductivity of the Y7C peptide reached 1.76 × 10^−2^ S cm^−1^, which is comparable to the values of other proton conducting materials and biomaterials (Supplementary Table 1). Y7C showed approximately 20 times higher proton conductivity than FFACAFF, which contains phenylalanine instead of tyrosine, suggesting a role of the phenolic hydroxyl group in proton conduction. This value is higher than that of other tyrosine-containing peptides, such as YYDCDYY and YYCYY (Supplementary Fig. 3).

### Proton-mediated resistive switching characteristics

**Fig. 2:**
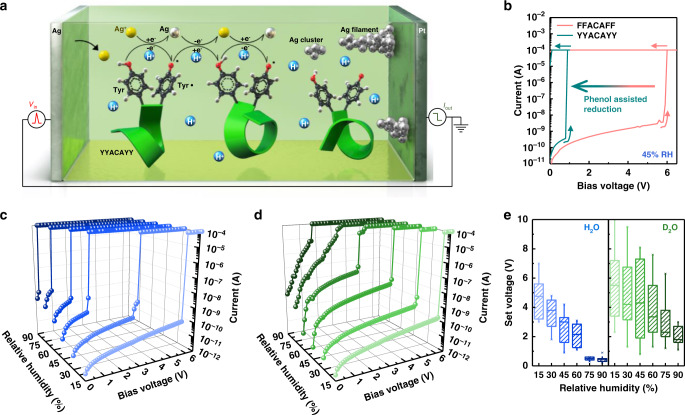
Proton-mediated resistive switching characteristics of the Y7C peptide. (**a**) Schematic diagram of proton-mediated resistive switching of the Y7C film. Helical peptide backbones are visualized as green ribbons. (**b**) Resistive switching characteristics of the Y7C peptide (emerald) and FFACAFF peptide (pink). Insets show the chemical structures of tyrosine and phenylalanine. (**c**, **d**) Resistive switching characteristics of Y7C under various relative humidities of H_2_O (**c**) and D_2_O (**d**) vapor. Increasing humidity is expressed in increasing darkness of colors. Each curve was measured after 2 h of fixed RH conditions. (**e**) Statistical box plots of the set voltage of the Y7C film as a function of the RH of H_2_O (left) and D_2_O (right). The interquartile range, mean, median, and outliers are represented by boxes, open squares, central lines, and whiskers, respectively.

Another interesting feature resulting from the high proton conductivity and redox capability of tyrosine is that the reduction and movement of silver ions inside the peptide film can be facilitated by both protons and applied electric fields (Supplementary Fig. 4). As a result, we discovered the proton-mediated resistive switching mechanism of Y7C film in a sandwiched structure between two electrodes (Fig. 2a and Methods). Highly insulating Y7C film in the pristine state becomes electrically conducting with the help of protons. When a positive voltage is applied, metal ions at the top electrode are generated and migrate to the bottom electrode, forming conducting filaments in the Y7C film via a mechanism similar to that of other resistive switching devices^25–33^. (Supplementary Note 2) However, a key difference is that under voltage bias, a phenolic hydroxyl group of tyrosine donates an electron to reduce Ag ions by generating a tyrosyl radical with the loss of one proton^34^. The resulting Ag atom transfers an electron to the neighboring tyrosyl radical, forming tyrosine. The coupled redox of Ag ions and tyrosine results in the migration of Ag atoms, the formation of Ag clusters, and alignment into Ag filaments. Therefore, a sufficient level of positively biased voltage, defined as a set voltage (V_set_ = 2.6 V at 45% RH), induces an abrupt and dramatic increase of more than six orders of magnitude in the current. The transition from a high-resistance state (HRS) to a low-resistance state (LRS) is clearly observed. A detailed illustration of the proton-mediated resistive switching mechanism is provided in Supplementary Note 3.

The involvement of silver ions was confirmed by the finding that replacing the silver electrode with gold completely nullified the memristor performance (Supplementary Fig. 5). To understand the role of tyrosine in the resistance transition of peptides, the FFACAFF peptide was introduced. The set voltage of the FFACAFF film was increased to 10.7 V compared with 1.8 V for the Y7C film (Fig. 2b and Supplementary Fig. 6). This verifies that the hydroxyl group in tyrosine reduces the energy barrier to the reduction of Ag ions and promotes the formation of a conduction path (Supplementary Fig. 7). These results indicate that the resistive switching characteristics of peptide films can be controlled by specific peptide sequences.

We examined the direct correlation between the resistive switching characteristic and the proton conduction of the peptide. The *I*–*V* characteristics of the peptide film under various RH conditions were obtained to verify the proton conduction effects by which the switching characteristic is modulated (Fig. 2c and Supplementary Note 4). Increasing RH induces a higher initial current at HRS by virtue of higher proton conduction. Moreover, the set voltage of the Y7C film decreased by more than one order of magnitude from 4.6 V to 0.4 V when RH increased from 15% to 90%. The changes in set voltage depending on various RH conditions are summarized in Fig. 2e. The switching endurance, temporal variation, spatial variation, and data retention under various RH conditions are summarized in Supplementary Fig. 8.

The kinetic isotope effect (KIE) was studied with deuterium oxide (D_2_O) vapor instead of H_2_O to determine direct evidence of the role of protons in facilitating resistive switching. Even with D_2_O vapor, resistive switching behaviors can also be shown. Showing a similar tendency, the set voltages decrease with increasing RH from 5.6 V at 15% to 2.1 V at 90% (Fig. 2d and Supplementary Fig. 9). However, the value of the set voltage with D_2_O is higher than that with H_2_O. For example, at RH 90%, the set voltage is 2.1 V with D_2_O but 0.4 V with H_2_O. Because the set voltage directly reflects the kinetic energy barrier, the higher value means that more energy is required for the heavier deuterium in the series of concerted processes involved in silver redox and ionic conduction. Therefore, this set of experiments led to the conclusion that protons are directly involved in the resistive switching mechanism. Based on this understanding of proton-dependent resistive switching characteristics, we demonstrate a memristor controlled bimodally and independently by either electron or proton inputs and simultaneously by both. To the best of our knowledge, there is no prior memory device operated only by ion-based stimuli such as calcium ions, sodium ions, or protons.

### Proton/electron controlled bimodal memristor

**Fig. 3:**
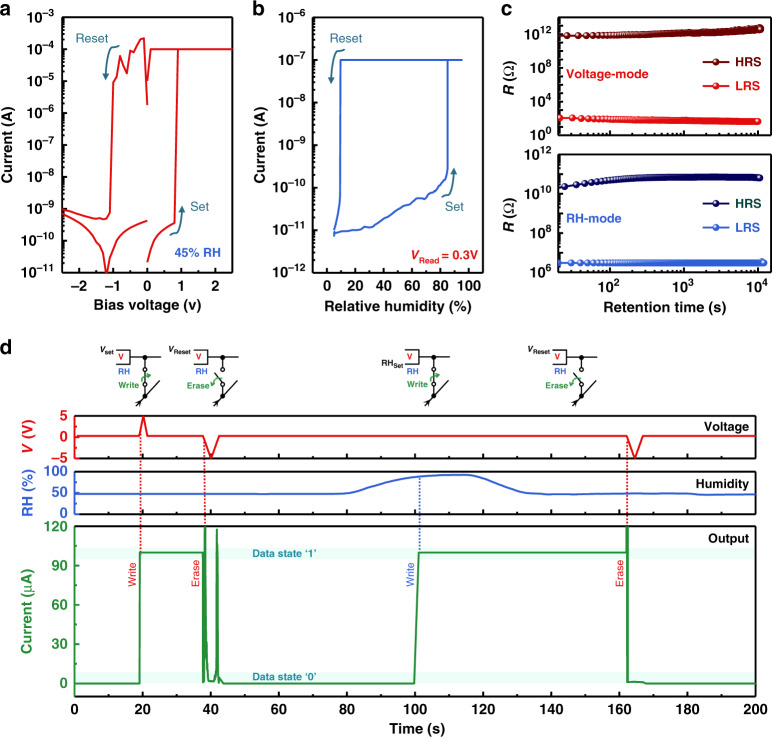
Proton/electron controlled bimodal memristor based on the Y7C peptide. (**a**) Current–voltage characteristics of the device at a relative humidity of 45%. The voltage sweep direction (0 → 2 → −2 → 0 V) is displayed with navy arrows. (**b**) Current humidity characteristics of the device with a constant read voltage of 0.3 V. The RH sweep direction (5 → 95 → 5%) is displayed with navy arrows. (**c**) Data retention characteristics of the device in voltage mode (top) and RH mode (bottom). (**d**) The current variation of the Y7C peptide memristor controlled by voltage and/or RH input, clearly showing bimodal operation. Voltage pulse, RH pulse, and current output are represented by red, blue and green curves, respectively.

The Y7C peptide film exhibits excellent resistive switching characteristics similar to other conventional memristors in with clear set and reset phenomena and a reasonable on/off current ratio of 10^6^ at 45% RH (Fig. 3a and Supplementary Fig. 10)^35–37^. Surprisingly, the resistive switching characteristics of the Y7C film were controlled only by RH change instead of electrical bias (Fig. 3b). RH was controlled by the injection of either N_2_ gas to reduce humidity or H_2_O humidified air to increase humidity and detected simultaneously by a humidity sensor (Supplementary Fig. 11 and Supplementary Note 5). With a constant read voltage of 0.3 V, a forward RH sweep from 5% to 95% was applied. The current abruptly increased by more than three orders of magnitude when RH reached ~90%, which corresponds to clear set operation to LRS (RH_set_). When a subsequent reverse RH sweep from 95% to 5% was applied, the current rapidly decreased at an RH of ~5%, corresponding to a clear reset operation to HRS (RH_reset_) (Supplementary Fig. 12 and Supplementary Note 6). Resistive switching of the Y7C film with an on/off ratio over 10^4^ is obviously controlled by RH alone. As the variation in RH leads to the change in proton conduction, resistive switching of the device is controlled by protons. The device retains its data states for over 10^4^ s regardless of whether it is written by voltage bias or by humidity (Fig. 3c).

As the peptide memristor operated by two individual modes, bias mode and humidity mode, was examined, the complementary bimodal operation of the peptide memristor was carried out (Fig. 3d). Because Ag redox for the Ag filament formation is coupled by the proton concentration and the switching by humidity is induced from the lowered onset potential, the LRS set by the humidity at low voltage bias can be erased by the electrical pulse. The data state of the peptide memristor was written by applying a positive voltage bias of 5 V and erased by a negative voltage bias of −5 V. Subsequently, the set operation was driven by increasing RH to 90%, and then the reset operation was successfully conducted by a negative voltage bias. Dual inputs using voltage and humidity, which correspond to electrons and protons, respectively, can complementarily write and erase data in one device (Supplementary Note 6 and 7).

### Proton-activated artificial synapses

**Fig. 4:**
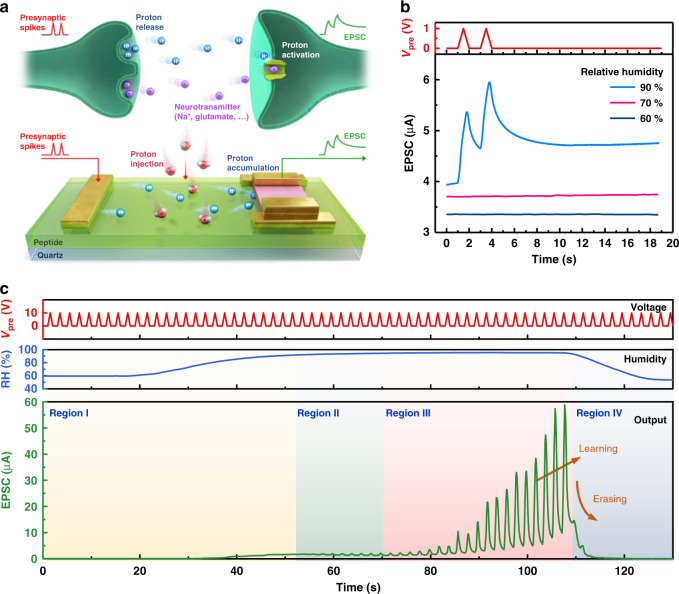
Proton-activated artificial synapses based on the Y7C peptide. (**a**) Schematics of the proton activation process in biological synapses (top) and artificial synapses (bottom). Presynaptic spikes and corresponding EPSCs are shown in red and green, respectively. In biological synapses, protons (blue) released from presynaptic neurons activate receptors at postsynaptic neurons to receive neurotransmitters (purple). (**b**) Postsynaptic currents of the artificial synapse under various RH conditions triggered by a pair of presynaptic voltage pulses (V = 1 v, ∆t = 1 s). PPF occurred at 90% RH (blue), while there was no presynaptic response at either 70% RH (magenta) or 60% RH (navy). (**c**) Proton-dependent plasticity of artificial synapses. The voltage pulse, RH pulse, and corresponding current output of the device are shown as red, blue, and green curves, respectively. Only when both voltage and RH inputs are activated does a notable learning process appear, and the synaptic response is suppressed drastically by reducing RH.

The concept of proton-activated devices can be further extended to short-term memory, synaptic transistors. Synaptic plasticity in terms of the ability of synapses to strengthen connectivity over time indicates that the learning process in the brain is not a unidirectional single-variable function from presynaptic inputs to postsynaptic current. It is a multivariable function in which the degree of learning is manipulated by interactions between a signaling agent and its environment. In particular, it has been proven that protons play modulatory roles that potentiate and/or inhibit neuronal ion channels^8^. Acid-sensing ion channels (ASICs), which detect extracellular proton concentration, function as signal receptors for proton signaling systems. In addition, the activities of other ion channels, including calcium, sodium, and potassium channels and glutamate and GABA receptors, are modulated by extracellular proton concentration. For instance, GluK2/K4 kainate receptors are potentiated by protons (Fig. 4a, top)^38^. In this regard, we expect that the peptide enables the proton activation process and regulation of the synaptic response.

For the fabrication of proton-controlled synaptic transistors, Y7C, In–Ga–Zn–O (IGZO), and Au were used for the gate dielectric, semiconductor, and metal, respectively (Fig. 4a, bottom and Methods). The Y7C film was spin-coated prior to the deposition of the channel and electrode layers for the in-plane-gate configuration. A lateral gate and IGZO channel were used for presynaptic terminal and postsynaptic neurons, respectively. The IGZO is used as the channel material due to its high on/off ratio, low leakage current, and process compatibility to the Y7C peptide film (Supplementary Note 8). Mobile protons in the Y7C electrolyte film play a role as the neurotransmitter^39^. The synaptic plasticity of the synaptic device was investigated by applying a presynaptic input voltage to the gate and measuring the excitatory postsynaptic current (EPSC) at the source/drain. Mimicking the proton-controlled regulation of biological neurotransmission, the synaptic response of the devices is activated under the protonic stimulus, and the plasticity can be precisely controlled. At low RH (under 80%), there was no drain current change when two consecutive triangular-shaped voltage pulses were applied to the gate (Fig. 4b). However, at RH values over 90%, which is sufficient for proton modulation, the synaptic response was activated due to the accumulation and depletion of protons in the hydrated peptide film (Supplementary Figs. 13 and 14). The drain current to a single pulse exhibited a typical EPSC response of biological synapses, showing a sudden increase and gradual decay depending on the spike duration (Supplementary Fig. 15a). When paired pulses were applied, the EPSC response to the second pulse was potentiated by 110% compared to the first response, representing paired-pulse facilitation (PPF) (Fig. 4b). The PPF index, defined as the ratio of the second peak to the first peak, is a function of the time interval between two pulses. The maximum PPF index was 151% with ∆t = 100 ms (Supplementary Fig. 15b). Data retention after stimulation, which is closely related to memory ability, was strengthened depending on the amplitude of stimulations, corresponding to the transition from short-term plasticity (STP) to long-term plasticity (LTP) of the biological synapse (Supplementary Fig. 15c). When the amplitude of the presynaptic voltage was in the range between 0.1 V and 2 V, the relaxation time was relatively short term within a few seconds. On the other hand, when the amplitude of the presynaptic voltage was larger than 2 V, the relaxation time increased to a few hundred seconds. (Supplementary Fig. 15d) In addition, spike-number dependent plasticity (SNDP) and spike-frequency dependent plasticity (SFDP) were observed by varying the stimulation number and the pulse frequency, respectively. When the number of stimulations increased from 2 to 20, the relaxation time of the data increased (Supplementary Fig. 15e). EPSC potentiation triggered by 10 pulses was enhanced by amounts ranging from 110% for the 1 Hz case to 2120% for the 10 Hz case (Supplementary Fig. 15f). The spatial and temporal variations of the activated artificial synapse are summarized in Supplementary Fig. 16 and Supplementary Note 9.

The time-scaled electrical characteristics of proton-dependent synaptic transistors were studied (Fig. 4c). Electrical presynaptic inputs with a pulse width of 1 s were sequentially applied while the modulatory RH input was varied. The proton-dependent plasticity in the postsynaptic current can be divided into 4 regions. In region I at low RH, EPSC was not affected by voltage input. When RH was increasing, EPSC started to increase because proton conduction increases with RH. In region II, the postsynaptic current started to respond to voltage inputs when RH exceeded 90%, while no sequential pulse facilitation was observed. Over time, a remarkable learning process appeared starting at 70 s at RH above 94% (in region III). In this high-RH region, the postsynaptic current was continuously potentiated as a presynaptic voltage pulse train was applied. When RH was decreased in region IV, the synaptic response was suppressed immediately, indicating that proton-induced synaptic connectivity was inhibited. This proton-activated synaptic plasticity occurred because proton accumulation and depletion at the interface of the Y7C film/IGZO took place as RH reached a threshold value of 94%. Accumulated protons resulted in the formation of an electric double layer (EDL), which showed a large capacitance and long-range lateral electrostatic coupling, in a similar mechanism as observed in other synaptic devices^40–45^. The slow relaxation resulting from low ionic mobility can induce learning and forgetting phenomena^46–49^. We further demonstrated the controllability of capacitive effects from proton accumulation by RH control so that the synaptic response could be activated and deactivated. The proton-sensitive feature of the Y7C peptide enables proton as a modulatory input to regulate whether to operate in the proton-gated transistors (Supplementary Note 10). Furthermore, our results are analogous to the multivariable functions of proton-sensitive neurotransmission, where synaptic learning behavior is affected by extracellular proton concentration.

## Discussion

In summary, our study demonstrates unprecedented multimodal neuron mimetic operations based on the control of proton activity in redox-active TRP films. The strategy developed here may embody new recent findings associated with biological reward-based learning, where the strength of synaptic connections is modulated. Furthermore, our observation emphasizes the importance of proton utilization in the reinforcing and switching of devices. We expect that this TRP film-based platform can be extended to other ions- and/or small molecule-based artificial sensory synapses on multiple timescales.

## Methods

### Circular dichroism analysis

For the CD analysis, a Y7C film was made via spin-coating a 1 wt% Y7C solution onto a quartz substrate. The CD spectrum of the Y7C film was recorded using a J-815 spectropolarimeter (Jasco) at 25 °C. The spectrum was collected from 260 to 180 nm with a 0.1 nm interval keeping the HT voltage less than 600 V for reliability. The data from 3 scans were averaged for each spectrum for reproducibility.

### Impedance analysis

Impedance analysis of the peptide films was carried out in the frequency range of 20 Hz to 1 MHz using an impedance analyzer (Keysight Technologies, E4990A). The real and imaginary impedance, magnitude, and phase angle were measured. An oscillator voltage of 500 mV and a DC bias voltage of 100 mV was applied.

### Fabrication of the memristor

A 10 nm Cr adhesion layer and a 100 nm Pt bottom electrode were first deposited on SiO_2_/Si substrate by direct current (DC) sputtering. A 2 wt% Y7C peptide solution was prepared by dissolving Y7C peptide powder (Beadtech, 95.08%) in trifluoroacetic acid (Daejung, 99.0%) at room temperature. The Y7C solution was spin-coated onto the substrates at 4000 rpm for 60 s, followed by Ag top electrode deposition by thermal evaporation at a pressure of ≈10^−6^ torr. The active area of 200 × 200 µm was defined by a shadow mask during the top electrode deposition. A homogeneous film with clear interfaces was confirmed by transmission electron microscopy (TEM) (Fig. 1c).

### Fabrication of the synaptic transistor

A 4 wt% Y7C peptide solution was spin-coated onto quartz substrates at 4000 rpm for 60 s. Then, 50-nm-thick In-Ga-Zn-O active layers were defined by a shadow mask and deposited by RF sputtering. The sputtering was performed at room temperature with a base pressure of <10^−6^ torr, an RF power of 100 W, and gas flow rates of 30 sccm and 0.5 sccm for Ar and O_2_, respectively. Then, 100-nm-thick Au with 10-nm-thick Ni as an adhesion layer defined by the shadow mask was thermally evaporated onto the channel layer (Fig. 4a).

## Supplementary information

Supplementary Information

## Data Availability

All data is available in the main text or the supplementary information.
