# Supplementary material for: Proton-enabled activation of peptide materials for biological bimodal memory
Source: Nat Commun. 2020 Nov 19;11:5896. doi: 10.1038/s41467-020-19750-5 (PMC7677316; doi:10.1038/s41467-020-19750-5)
Supplement: Supplementary file 1 — Supplementary Information [file 41467_2020_19750_MOESM1_ESM.pdf]

Supplementary Information

Proton-Enabled Activation of Peptide Materials for Biological Bimodal Memory

Min-Kyu Song[1], Seok Daniel Namgung[2], Daehwan Choi[1], Hyeohn Kim[2], Hongmin Seo[2], Misong Ju[2], Yoon Ho Lee[2], Taehoon Sung[1], Yoon-Sik Lee[3], Ki Tae Nam[2] and Jang-Yeon Kwon[1]*.*

1 School of Integrated Technology, Yonsei University, Incheon 21983, Republic of Korea.
2 Department of Materials Science and Engineering, Seoul National University, Seoul 08826, Republic of Korea.
3 School of Chemical and Biological Engineering, Nano Systems Institute, Seoul National University, Seoul 08826, Republic of Korea.

*** To whom correspondence should be addressed:**

Ki Tae Nam, Ph.D.
Department of Materials Science and Engineering
Seoul National University
Seoul 08826, Korea (Republic of)
Tel: +82-2-880-7094
E-mail: nkitae@snu.ac.kr

Jang-Yeon Kwon, Ph.D.
School of Integrated Technology
Yonsei University
Incheon 21983, Korea (Republic of)
Tel: +82-32-749-5837
E-mail: jangyeon@yonsei.ac.kr

Supplementary Notes

Supplementary Note 1. Proton conductivity calculation

The proton conductivity of the peptide film was calculated by fitting the data of the Nyquist plot acquired from the impedance spectroscopy measurement. The Nyquist plot showed a semicircle in the high-frequency region and an inclined tail in the low-frequency region which agrees with the typical behavior of proton conductors (Fig. 1e and Supplementary Fig. 2, 3)[1-3]. The semicircle represents the bulk impedance and the tail represents the accumulation of protons at the film/electrode interface. Estimated values of bulk resistance were derived from the diameters of the semicircles by fitting the plot to an RC equivalent circuit that has been to model proton exchange membranes (Supplementary Fig. 1)[4-6]. Thus, the proton conductivity of the films can be calculated from the following equation.

$$\sigma = \frac{l}{AR_b}, \quad (1)$$

where σ, l, R_b and A are conductivity, film thickness, bulk resistance and area of the top electrode, respectively.

Supplementary Note 2. Resistive switching characteristics study

The role of tyrosine in the resistive switching of Y7C is depicted in Fig. 2a. Tyrosine in the spin-coated Y7C solution in trifluoroacetic acid remains protonated because the pK_a of the phenol group in tyrosine is approximately 10.1[7]. Under a voltage bias, tyrosine actively renders an electron to reduce the Ag ion from the top electrode and a proton, generating a tyrosyl radical. The reduced Ag atom is unstable due to the negative reduction potential of -1.8 V and is easily oxidized back to an Ag ion[8,9]. Thus, the Ag atom transfers an electron to the tyrosyl radical, forming protonated tyrosine. This reaction continues until the Ag ion contacts the Pt electrode and/or forms an Ag cluster, because the oxidation of Ag becomes exergonic (+0.8 V) when Ag atoms aggregate[9,10]. Through this process, tyrosine in Y7C promotes the Ag redox and migration so that the set voltage of Y7C is much smaller than that of F7C (Fig. 2b).

The Y7C memristor shows not only bipolar resistive switching characteristics but also unipolar resistive switching characteristics so that the polarity of the reset voltage can be either positive or negative (Supplementary Fig. 10a). As observed in the bipolar case (Fig. 3a, c), the Y7C memristor also exhibits excellent cycle endurance and data stability in the unipolar case (Supplementary Fig. 10b, c). The set process was conducted with a current compliance level of 10^{-4} A to avoid the permanent breakdown of the device. To investigate the conduction

mechanism of the Y7C memristor, double-logarithmic fitting of the I-V characteristics was carried out (Supplementary Fig. 10d). The slope of 1 in the HRS and LRS region indicates ohmic conduction behavior, which can be described using the following equation:

$$J_{Ohm} = q n_0 \mu \frac{V}{d}, \quad (2)$$

where J_{Ohm}, q, n_0, μ, V, and d are the current density due to Ohm's law, intrinsic charge density, charge mobility, applied voltage, and film thickness, respectively. The slope changed from 1 to 2 in the high-voltage region in the HRS. In this region, the charge conduction mechanism can be explained by the Mott-Gurney law, which represents the trap-controlled space charge limited current, known as SCLC, using the following equation[11]:

$$J_{Mott} = \frac{9}{8} \mu \varepsilon \frac{V^2}{d^3}, \quad (3)$$

where J_{Mott} and ε are the current density due to the Mott-Gurney law and dielectric constant, respectively.

To confirm the origins of the conduction path in the peptide film, we verified the oxidation effect of the top electrode material on resistive switching by introducing other electrodes in place of the Ag electrode. No resistive switching was observed even at a bias voltage of 35 V with the structure of Au/Y7C/Pt (Supplementary Fig. 5a). This means that the Y7C peptide is not able to oxidize either electrode due to the stable nature of Au and Pt and remains highly insulating even under a high electric field. Thus, Ag redox evidently plays a key role in the resistive switching phenomenon in accordance with the previously reported electrochemical metallization (ECM)[11-15]. Furthermore, the Y7C film shows resistive characteristics with other redox active electrodes, including Cu and Mg (Supplementary Fig. 5b, c). Despite the relatively unstable performance of the Y7C memristors, several cycles of resistive switching in the cases of Cu and Mg were observed (Supplementary Fig. 5d, e).

The distribution of the set voltages and the durability under various RH conditions are shown in Supplementary Fig. 8. Each row corresponds to the device characteristics under the fixed RH conditions in which the devices were kept for at least 2 hours. The first column shows the RH levels for each column. The second column presents the resistance states from 100 cycle tests. Although there are considerable fluctuations of the resistance caused by incomplete set and reset, the HRSs and the LRSs can be distinguished at all RH conditions. Endurance of the Y7C peptide memristor should be further optimized for repetitive write and erase operation. The third and fourth columns show the temporal and spatial distributions of the set voltages, respectively. The temporal and spatial variations are defined as the standard deviation divided

by the average of the set voltages (σ/μ). These results are in accordance with Fig. 2e that the set voltage is reduced with increasing RH. The fifth column displays the retention tests of the HRSs and the LRSs for 10^4 s. The data retention of the Y7C peptide memristor remains quite stable even under various RH conditions. The durability and variation of the device still need to be improved to show stable and repetitive performance and be built into a large-scale crossbar array. However, the reproducible phenomenon of proton-mediated switching across the numerous devices shows the distinct and novel properties of the Y7C peptide memristor.

Supplementary Note 3. Proton-mediated switching mechanism

How proton is involved in the resistive switching can be explained in following four steps with experimental evidences.

1. Origin of the resistive switching

Similar to the conventional electrochemical metallization (ECM) memories[16], oxidation of the metal electrode causes the resistive switching of the Y7C peptide memristor. The involvement of silver redox was confirmed by the finding that replacing the silver electrode with gold completely nullified the memristor performance (Supplementary Fig. 5). Under the voltage bias, Ag top electrode is oxidized and dissolved into the peptide layer, and finally forms Ag filament connecting top and bottom electrodes.

2. Role of tyrosine in the resistive switching

Tyrosine is known as a redox-active amino acid that plays a key role in biological processes[17]. We have shown that the tyrosine rich Y7C peptide has catalytic property owing to low oxidation potential in the previous work[18]. In this regard, we assumed that tyrosine plays a key role in redox reaction with Ag atoms resulting resistive switching in the Y7C film. To understand the role of tyrosine in the resistance transition of the peptide film, the FFACAFF peptide which contains phenylalanine (F) instead of tyrosine was introduced. The only difference between two similar amino acids is that tyrosine has phenolic hydroxyl group while phenylalanine does not. The set voltage of the FFACAFF film was increased to 10.7 V compared with 1.8 V for the Y7C film (Fig. 2b and Supplementary Fig. 6). This indicates that the hydroxyl group in tyrosine is involved in the resistive switching which results from the redox of Ag atoms and the formation of a conduction path.

The redox chemistry of tyrosine is inherently proton-coupled, indicating that electron transfer is accompanied by proton releasing and accepting[19]. Therefore, it can be assumed that the promotion of Ag filament formation with phenolic hydroxyl group is because reduction of

Ag ions is dominated by charge transfer from phenolic hydroxyl group in tyrosine to Ag ions resulting in deprotonation of tyrosyl radical.

3. Proton involvement in the redox reaction

Proton involvement in charge transfer between tyrosine and Ag atoms is also verified by the experiment on kinetic isotope effect of hydrogen. Deuterium, the isotope of hydrogen and known as heavy hydrogen, has approximately twice mass of hydrogen. To investigate hydrogen ion involvement, water vapor is replaced by deuterium oxide vapor and the kinetics of the phenomena is compared. After removal of water vapor in the chamber, deuterium oxide (D_2O) vapor with nitrogen carrier gas was injected to adjusting RH value. The set voltages in which the transition from HRS to LRS occurs were measured for each RH condition ranging from 15 % to 90 %. As shown in Supplementary Fig. 9a, the set voltages in D_2O condition is higher than that in H_2O condition at the same level of 90 % RH. In addition, Supplementary Fig 9b, presents the linear relationship between the set voltages and the RH. The slope of the fitted curve for the D_2O case is 1.25 times higher than that for the H_2O case. This suggests that the difference in the atomic mass between hydrogen ion and deuterium ion induces the changes in the kinetics of the redox reaction between Ag atoms and tyrosine. Therefore, the resistive switching phenomenon of the Y7C peptide memristor is enhanced by proton-involved Ag redox by tyrosine.

In addition to the kinetic isotope experiment, the comparative study on the effect of air between tyrosine and phenylalanine was carried out in this revision. Supplementary Fig. 6 shows the I-V characteristics for the Y7C peptide and FFACAFF peptide of the set process both in vacuum and ambient conditions. The set voltage of the Y7C peptide memristor exhibited 57 % reduction after the devices were taken out of vacuum, while that of the FFACAFF peptide memristor showed relatively low change of 5 %. This result is accordance with the assumption that Ag redox is promoted by proton-coupled charge transfer of tyrosine (Supplementary Fig. 7).

4. Redox-associated proton conduction in the Y7C film

The Y7C peptide shows the high proton conduction which depends exponentially on the external humidity (Fig. 1f). Comparison of the proton conductivity between YYACAYY (Y7C) and FFACAFF verifies that the existence of phenolic hydroxyl group in tyrosine plays a critical role in the proton conduction (Supplementary Fig. 3). This corresponds to our previous findings that the tyrosine rich Y7C peptide shows proton coupled electron transfer (PCET) even in thick film that plays important role in the high proton conduction from the measurement of Onsanger

coefficient[20]. These results suggest that phenolic hydroxyl group acts as a hopping site for proton. Thus, it can be assumed that the electron transfer is coupled with the protonation and deprotonation of tyrosine during proton hopping. The proposed redox-associated conduction is highly probable due to the low oxidation potential of the Y7C peptide of 0.86 V as shown in the cyclic voltammetry (Supplementary Fig. 4). This electrochemical reactive property of the Y7C peptide leads the acceleration of reduction of Ag ions as a consequence of the oxidation of the Y7C peptide. As a result, we propose the proton-mediated resistive switching mechanism that increasing humidity induces the increased proton conduction in the Y7C peptide resulting the promotion of the reduction of Ag ions.

Supplementary Note 4. Relative humidity control (steady state)

Each humidity condition was controlled by an injection of either N_2 gas to reduce the humidity or H_2O humidified air to increase the humidity and detected by a humidity sensor kit (Sensirion, SHT31). To verify the minimum exposure time for proton saturation in the peptide film, the current of the peptide film was measured with various relative humidities (RHs) and incubation times. After 1 hour of exposure to 60 % RH, the current of the peptide film increased approximately 10 times higher than the initial current of the film exposed to ambient conditions at 30 % RH over 1 day. After 2 hours of exposure, the current further increased by a factor of 4. The increase in the current saturated after 2 hours of exposure to 60 % RH, as no increase in the current was observed in the case of exposure for 3 hours. For the 80 % RH condition, the increase in the current saturated even after 2 hours of exposure. Thus, the devices were kept in the chamber for 2 hours at each RH condition, which is considered to be the minimum incubation time to allow moisture to diffuse in the peptide film.

Supplementary Note 5. Relative humidity control (dynamic)

A dynamic measurement of the RH value was conducted at a frequency of 2 Hz by a digital humidity sensor (Sensirion, SHTC3). A dynamic RH sensor was placed adjacent to the device to measure the RH variation on the surface of the peptide film (Supplementary Fig. 11a, b). Due to the change in the proton conduction, the transient current changes simultaneously as RH changes (Supplementary Fig. 11c). Prior to the RH sweep for the humidity mode of the Y7C memristor, the RH value was decreased to an extremely low value of 5 % by injecting N_2 gas into the chamber. The pressure and temperature were kept at 760 Torr and 21±1 ºC, respectively, during the RH sweep. A forward RH sweep from 5 % to 95 % was applied by

injecting humidified air. The RH rise time varies from ~0.5 min to ~20 min. At an extremely high RH corresponding to the set humidity regardless of the rise rate, abrupt resistive switching from the HRS to the LRS was observed (Supplementary Fig. 12a, b, 17a). A reverse RH sweep from 95 % to 5 % was applied by injecting N_2 gas. The fall time varies from 60 s to 120 s. At an extremely low RH corresponding to the reset humidity, regardless of the fall rate, abrupt resistive switching from the LRS to the HRS was observed (Supplementary Fig. 12b, 17b). The scan rates are defined as follows:

$$\tau_{rise} = \frac{RH_{set} - RH_{initial}}{t_{set} - t_{initial}}, \tau_{fall} = \frac{95\ \% - RH_{reset}}{t_{reset} - t_{RH=95\%}}, \tag{4}$$

where τ_{rise}, τ_{fall}, RH_{set} and RH_{reset} are the rise rate, fall rate, set humidity, and reset humidity, respectively. The set humidity is defined as the RH value when the current exceeds 90 % of the current compliance level only when nonvolatile resistive switching is observed. The reset humidity is defined as the RH value when the current is measured below 0.1 nA.

Supplementary Note 6. Humidity mode of the Y7C memristor

As the humidity increases, the transient current increases and the Ag redox is promoted due to accelerated PCET[4,17,21]. Therefore, applying humidity without any changes in the voltage bias induces abrupt resistive switching. Regardless of the read voltage from 0.3 V to 1.2 V, resistive switching occurred at approximately 90 % RH (Supplementary Fig. 18). In hydrated Y7C at a high RH, the major parameter affecting the formation of a conducting filament is humidity, not the voltage bias. The reset humidity is a function of the current compliance level during the measurement (Supplementary Fig. 19). When the current compliance level was set to 10^{-7} A, the reset operation occurred at 16 % RH. On the other hand, the reset humidity became 47 % RH when the current compliance level was set to 10^{-8} A. This means that the set/reset window changes according to the current compliance level setting during the RH sweep. The stability of the Ag conduction path might decrease while the current compliance decreases[22]. In this regard, the set/reset window of the humidity mode can be adjusted as needed.

Metal oxidation effects on resistive switching were also studied for the humidity mode of the Y7C memristor. No resistive switching even at 95 % RH was observed with the Au electrode as the top electrode (Supplementary Fig. 20). Only an increase in the transient current was observed under various read voltage biases. This result is in accordance with the bias mode of the Y7C memristor.

Supplementary Note 7. Bimodal operation of the Y7C peptide memristor.

The proton mediated switching and the electron mediated switching are not separated mechanism. Since proton and electron are coupled in charge transfer between tyrosine and Ag ions, the mechanisms of resistive switching in two modes are basically the same and, thus, resistive switching in the peptide can be controlled either by bias or humidity input. When the peptide is in the high-resistance state (HRS), charge conduction in the peptide film is proton-coupled electron transfer which can be observed in the impedance analysis shown in Fig. 1e. When the peptide is in the set process either by bias mode and humidity mode, both proton and electron are responsible in the switching phenomenon. In the case of the Y7C peptide, proton acts as a dominant factor of the switching because of proton-coupled charge transfer property of tyrosine. However, the charge conduction at the low-resistance state (LRS) is solely caused by electron conduction. It is because the conduction between two electrodes in the LRS is through the silver filament in the peptide film. Once set process is done, the carrier of the conduction is dominated by electrons. Therefore, the written or erased data from two different switching modes cannot be distinguished theoretically if the other conditions remain the same.

However, distinct states of LRS can be realized by exploiting the experimental result that the current compliance level windows during the set process for stable operation are different. The resistance of conductive filament formed after the set operation depends on the set condition including the current compliance level, read voltage, and RH value. It is reported that the size of metal filament is tuned by the compliance level causing difference in the resistance[14,23,24]. Therefore, we examined dependency of the conductance of the LRS (Supplementary Fig. 21) and dependency of set and reset humidity on the current compliance level (Supplementary Fig. 19). Reset humidity depends on the compliance level because the stability of the conductive filament is changed with the size of the filament (Supplementary Note 6).

Based on the result, the compliance level of the humidity mode is set to be 10^{-7} A while that of bias mode ranges from 10^{-4} A to 10^{-7} A. For example, if the resistance of LRS set by two different mode should be distinguished, the compliance levels of the bias mode and the humidity mode can be set as 10^{-4} A and 10-7 A, respectively. Therefore, we can distinguish between the two modes in which resistive switching from HRS to LRS have done by measuring the resistance of LRS. Supplementary Fig. 22 displays bimodal operation of the Y7C peptide memristor. Current compliance level was set to 10^{-4} A and 10^{-7} A for bias mode and humidity

mode, respectively. The conductance level indicated as the state ‘0’ means the pristine state (or HRS) of the Y7C film which is very low (≈10^{-11} S). After the set operation in the bias mode, the conductance level is immediately changed to the state ‘2’ corresponding to the electrically set LRS. The state is returned to the initial state by reset process in the bias mode. Following RH sweep induces the increase in the conductance and the set operation at high RH. In this case, the conductance level is increased to the state ‘1’ corresponding to the LRS set by humidity. Two states of ‘1’ and ‘2’ can be distinguished easily by measuring the conductance of the Y7C film.

Supplementary Note 8. Advantages of In-Ga-Zn-O semiconductor for artificial synapse

We utilized IGZO as a semiconductor material because IGZO has several advantages of physical performance and process compatibility. First, IGZO-based transistors exhibit relatively high on/off current ratio due to the intrinsic high mobility and low carrier concentration[25]. As RH increases, proton conductivity of the Y7C peptide increases significantly. Thus, leakage current from gate electrode would be increased and cover the drain current modulation. To clarify the carrier modulation from the lateral gate, the ratio between on current and off current should be large enough. However, if the transistor operates in depletion mode, the energy dissipation of artificial neural network based on the transistor will be seriously increased. Since IGZO semiconductor has very low intrinsic electron concentration, the transistors based on IGZO operate in enhancement mode. Compared to the other oxide semiconductor materials, IGZO still exhibits very low off current showing high on/off ratio. Therefore, the energy dissipation of the artificial neural network based on the transistors will be potentially decreased. Owing to these electrical properties, there have been several attempts to utilize IGZO as a channel material of synaptic transistors[26,27]. In addition to this electrical advantages, physical properties of IGZO including fair flexibility and good transparency are also preferable to be applied in bio-integrated systems. We previously reported the bio-implantable devices based on the peptide and have developed the photon sensitive oxide-peptide complex for future works. Therefore, the physical properties of IGZO film as well as the electrical properties are adequate for our research scope.

Furthermore, process compatibility of IGZO to the peptide is also considered. One of the process barriers of the peptide film is that the peptide film is easily dissolved by liquid and degraded by thermal stimulus. For that reason, deposition on the peptide film is limited to room-temperature and liquid-free process. To fabricate the lateral gating transistors on the

peptide film, semiconductor layer is deposited on the peptide film. IGZO can be deposited by room-temperature sputtering without performance compensation. For that reason, IGZO can be deposited directly on the peptide film. Also, sputtered IGZO shows good uniformity that can be advantageous to be applied in a large-scale crossbar array. IGZO exhibits not only adequate electrical performance for artificial synapse but also process compatibility preventing degradation of the peptide film.

Supplementary Note 9. Proton-activated artificial synaptic plasticity

Synaptic plasticity was emulated by applying presynaptic spikes to the proton-activated artificial synapse at 90 % RH. Triangular-shaped presynaptic spikes were applied to the lateral gate of the device, and the EPSC was measured between two electrodes on the top of the channel layer (Fig. 4a, bottom). Analogous to a biological synapse, the EPSC was modulated by the stimulation time, which corresponds to spike duration-dependent plasticity (Supplementary Fig. 15a). As the stimulation time increased from 100 ms to 1000 ms, the amplitude and retention time of the EPSC response increased. Two consecutive presynaptic spikes caused a transient synaptic enhancement referred to as paired-pulse facilitation (PPF), which is a function of the time interval between the spikes (Supplementary Fig. 15b, inset). In this case, the amplitude and duration of the spikes were fixed at 1 V and 1 s, respectively. The PPF index decayed as the time interval of the spikes increased (Supplementary Fig. 15b). After being stimulated by postsynaptic spikes, the EPSC underwent a decaying process, which is referred to as the relaxation process of the short-term potentiation of the synapse. The data loss of the device can be described as an exponential decay function called the Ebbinghaus forgetting curve[28]:

$$I = I_0 + I_A e^{-\frac{t}{\tau}}, \quad (5)$$

where I_0, I_A, t and τ are the current offset, fit constant, time after the stimulation, and relaxation time constant, respectively. Data retention as a function of amplitude of presynaptic voltage was measured after single stimulation with duration of 1 s. (Supplementary Fig.15c) Transition from short-term plasticity (STP) to long-term plasticity (LTP) is observed when presynaptic voltage is larger than 2 V. (Supplementary Fig. 15d) To observe the relationship between the stimulation number and relaxation time constant, various numbers of presynaptic spikes with a fixed amplitude of 1 V and a duration of 1 s were applied. The relaxation time constant was increased with a larger number of stimulations (Supplementary Fig. 15e).

Frequency modulation of the EPSC was observed by applying 10 consecutive spikes with a fixed amplitude of 1 V and a duration of 100 ms (Supplementary Fig. 15f). This indicates that the spike-frequency-dependent plasticity of a biological synapse is emulated by the peptide-based synaptic device.

Spatial variation of humidity effects on the carrier modulation was identified in various RH conditions. (Supplementary Fig. 16a) On and off current of the transfer characteristics was measured at Vg = 20 V and -5 V, respectively. No current modulation from gate voltage occurred at 60 % and 70 % RH. Only at 90 % RH, all the devices showed carrier modulation by gate voltage despite the considerable spatial variation. The spatial variations of on current, off current and on/off ratio were 137.6 %, 98.3 % and 145 %, respectively. Due to the unwilling spatial variation of the transfer characteristics, the analog switching properties of the devices also exhibited the low uniformity as shown in Supplementary Fig. 16b. The peak conductance after 30 pulses of potentiation showed the spatial variation of 76.4 %.

Temporal variation of the artificial synapse based on the Y7C peptide is also shown in Supplementary Fig. 16. The on and off currents from 20 cycles of transfer curves were measured at gate voltage of 20 V and -5 V, respectively (Supplementary Fig. 16c). The temporal variations of the on and off current were 35.5 % and 15.8 %, respectively. Voltage sweep ranged from -30 V to 30 V and it probably degraded the transfer characteristics because potentiation/depression curve shown in Supplementary Fig. 16d does not exhibit significant degradation during 10 cycles. The temporal variation of the maximum conductance was 5.17 %. The temporal variation of the device is much better than the spatial variation meaning that the device operate stable despite low uniformity.

Supplementary Note 10. Lateral long-range electrostatic effect at high RH.

In the high enough humidity condition, thin and homogeneous layer of water can be formed on the device. Thus, we fabricated IGZO transistors without peptide layer for a comparative study on gating effect of the peptide at high RH. IGZO channel and Au electrodes were deposited directly on the quartz substrate for the device without peptide layer. Supplementary Fig. 23a shows the transfer curves of the devices with and without peptide layer at 90 % RH. Gating effect in the device with the peptide layer was clearly observed while that in the device without the peptide layer was not observed in the range of Vg = ± 30V. On current of the device with peptide layer is significantly increased only at 90 % RH. In addition, capacitances with and without the peptide layer were measured. Capacitance-voltage (C-V)

characteristics is displayed in Supplementary Fig. 23b. Even though some fluctuation was observed, capacitance of the device with the peptide layer was measured to be approximately 2pF at 90 % RH while only noise was observed at 70 % and 80 % RH, not capacitance. In the case of the device without the peptide layer, capacitance was not observed even at 90 % RH. This indicates that the high humidity does not solely induce the electrostatic coupling on the semiconductor region for carrier generation. Lateral gating effect from electro hydrolysis did not occur probably due to the long distance of 100 μm from the channel to the gate. In addition, typical capacitance-frequency (C-f) characteristics of ion gating that capacitance increased as frequency decreased was measured in the case of the device with the peptide layer at 90 % RH (Supplementary Fig. 23c)[29]. This suggests that the capacitive effect in high RH is owing to the accumulation of ions, most likely protons.

Supplementary Figures

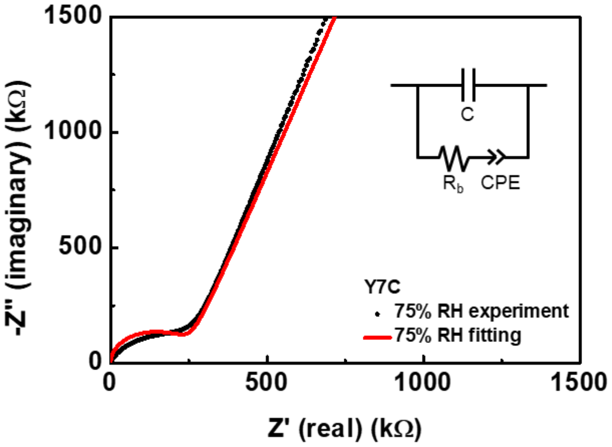

Supplementary Figure 1 | Impedance analysis of peptide. Nyquist plot (black) and the fitting result (red) from the Y7C peptide with Au electrodes at 75 % RH. The circuit diagram represents the equivalent circuit consisting of the bulk resistance (R_b), constant phase element (CPE) and parallel capacitive term (C) indicating a distributed capacitive contribution of the network (inset)[4,5,30].

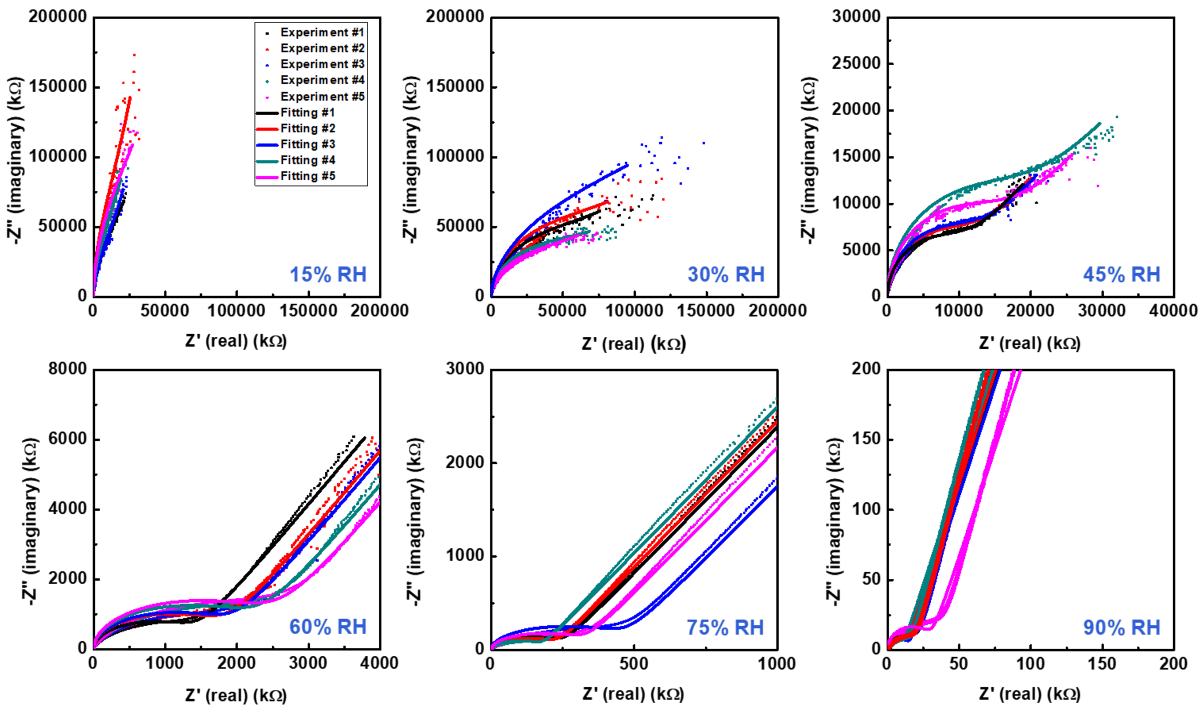

Supplementary Figure 2 | Nyquist plots of the experimental data (scatter) and the fitting results (solid) under various RH conditions. Each data point and the corresponding fitting line are displayed in the same color. At low RHs of 15 % and 30 %, the fitting curves do not show semicircles and tails because of the large diameter of the semicircles corresponding to high bulk resistance, but can be inferred to be in accordance with other curves at high RHs. Five samples at each RH condition were measured after 2 hours of exposure to the RH condition.

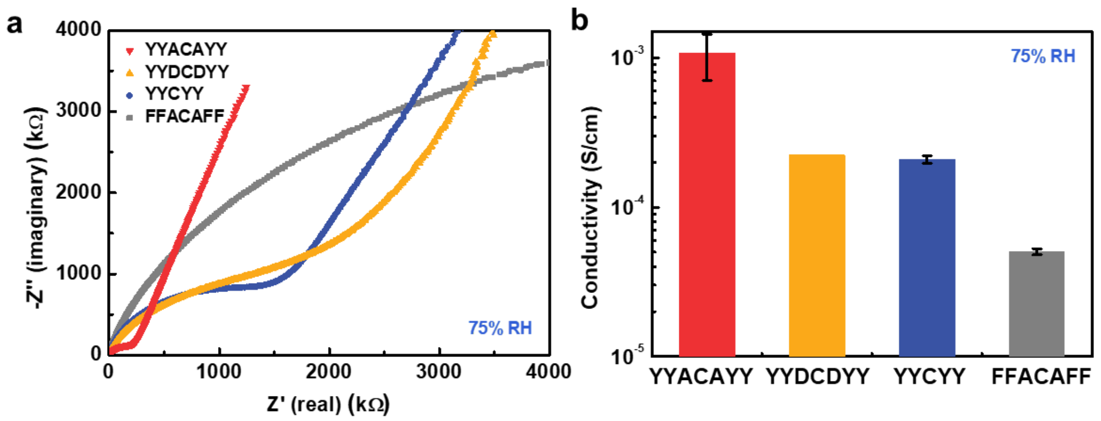

Supplementary Figure 3 | Proton conductivity comparison among peptides with different sequences. a, Nyquist plots of various peptides measured at 75 % RH. Four different peptides were investigated, and all showed semicircles and tails. Y, A, C, D and F represent one letter symbols of tyrosine, alanine, cysteine, aspartic acid and phenylalanine, respectively. **b,** Calculated proton conductivity of various peptides. The lateral dimension of the Au electrode (200 µm x 200 µm) was fixed, and the thickness of each film was measured by an alpha step (YYDCDYY = 166 nm, YYCYY = 124 nm and FFACAFF 142 nm). Each peptide is plotted in the same color in (A) and (B). The error bars represent the mean ± SD.

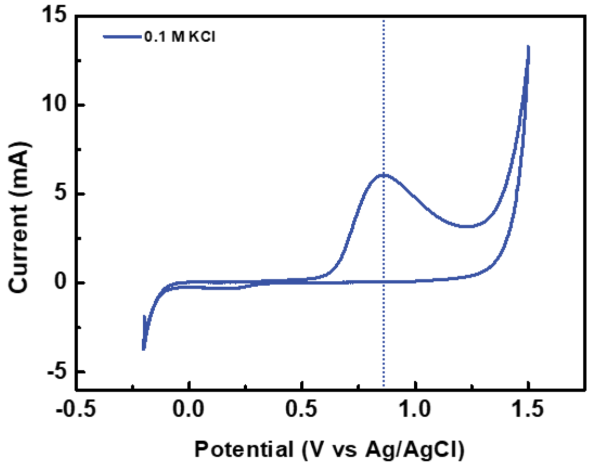

Supplementary Figure 4 | Cyclic voltammetry of the Y7C peptide on FTO glass in 0.1 M KCl electrolyte. The electrochemical oxidation of tyrosine (Tyr→Tyr·) resulted in the peak at 0.86 V versus the Ag/AgCl electrode

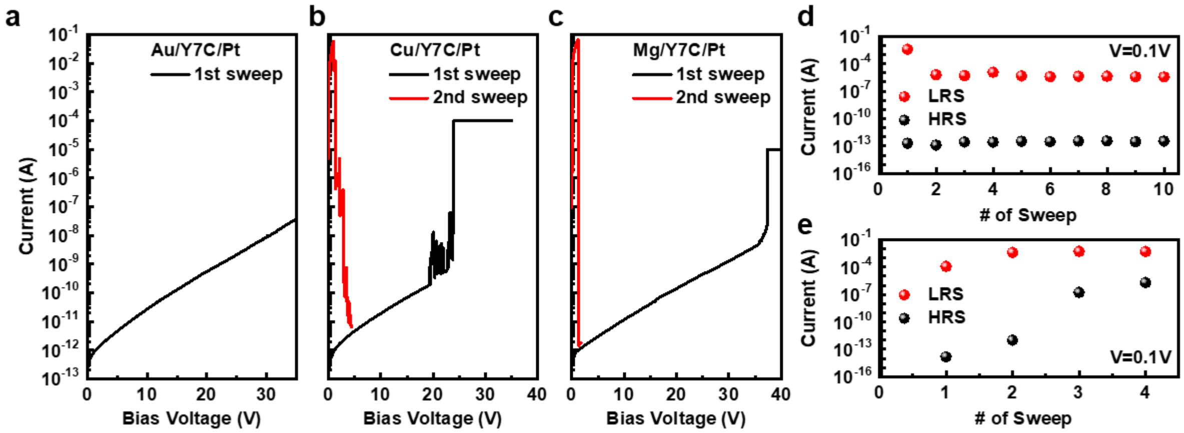

Supplementary Figure 5 | Effect of metal oxidation on the resistive switching characteristics of the Y7C film. a-c, Resistive switching characteristics of peptide memristors with (**a**) Au, (**b**) Cu, and (**c**) Mg as the top electrode. Resistive switching was not observed with the Au electrode, while unipolar resistive switching was observed with the Cu and Mg electrodes. The black and red curves indicate the first set and first reset process, respectively. **d-e,** Cycle endurance on which currents of the LRS and HRS under a read voltage of 0.1 V are plotted. Ten cycles of resistive switching occurred in the case of (**a**) the Cu electrode, while 4 cycles of resistive switching occurred in the case of (**b**) the Mg electrode.

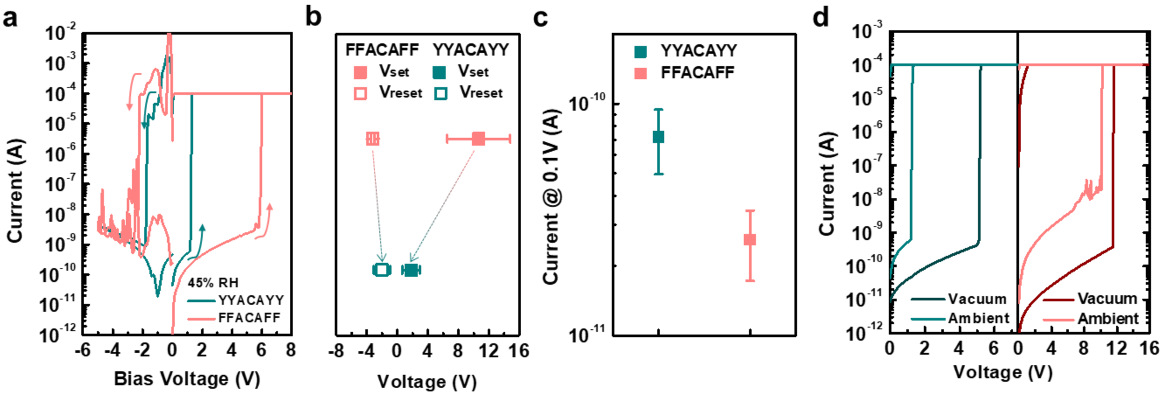

Supplementary Figure 6 | Resistive switching characteristics and device parameters of the YYACAYY and FFACAFF memristors. a, IV characteristics of the YYACAYY and FFACAFF films at 45 % RH. Both curves show the set process in the positive sweep and the reset process in the negative sweep, which are indicated by arrows. **b,** Set (solid square) and reset (open square) voltages of the YYACAYY and FFACAFF films. The set/reset window of the YYACAYY film is obviously narrower than that of the FFACAFF film. The set and reset voltages were reduced by 83 % and 37 %, respectively, by replacing phenylalanine with tyrosine. **c,** Off current of the YYACAYY and FFACAFF films under a voltage bias of 0.1 V. Pristine FFACAFF is more insulative than YYACAYY, in accordance with Supplementary Fig. 3. The data of YYACAYY and FFACAFF in (**a-c**) are shown in emerald and pink, respectively. The error bars represent the mean ± SD. **d,** Comparison of the set voltages in vacuum and ambient between YYACAYY and FFACAFF. I-V characteristics of the memristors based on the YYACAYY peptide (left) and FFACAFF peptide (right) in vacuum and ambient condition.

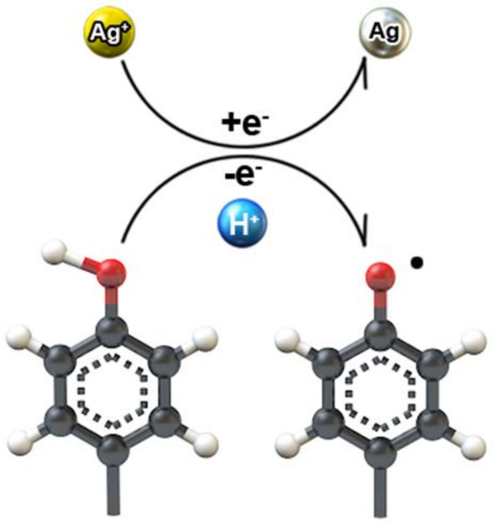

Supplementary Figure 7 | Charge transfer between tyrosine and silver. Silver ions are reduced to silver atoms during deprotonation of tyrosine. Tyrosine renders one electron and one proton during the deprotonation.

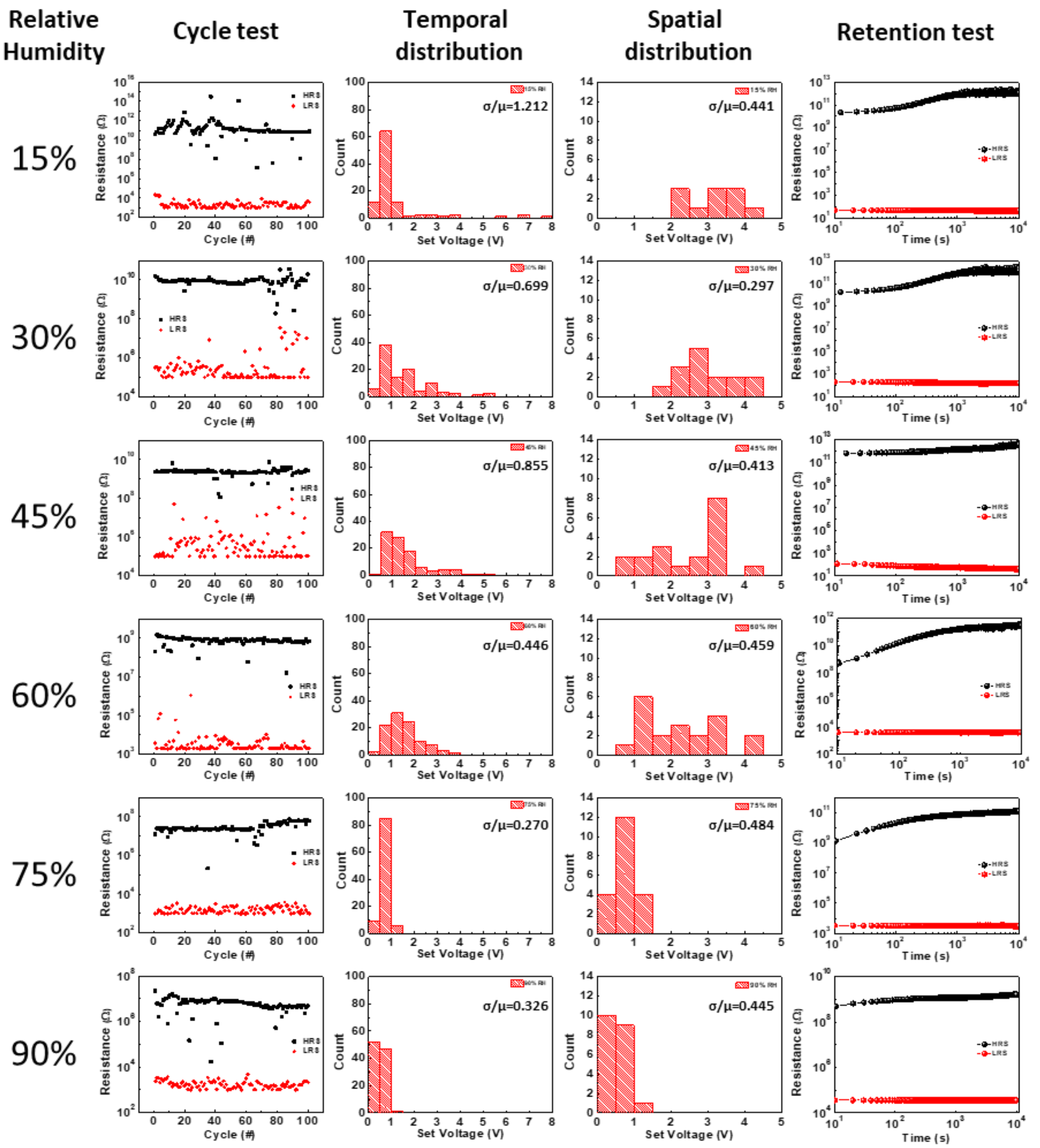

Supplementary Figure 8 | Device performance of the Y7C peptide memristor under various RH conditions. Each row represents the same RH conditions. Each column displays cycle test (2nd column), temporal variation (3rd column), spatial variation (4th column) and retention test (5th column) of the device.

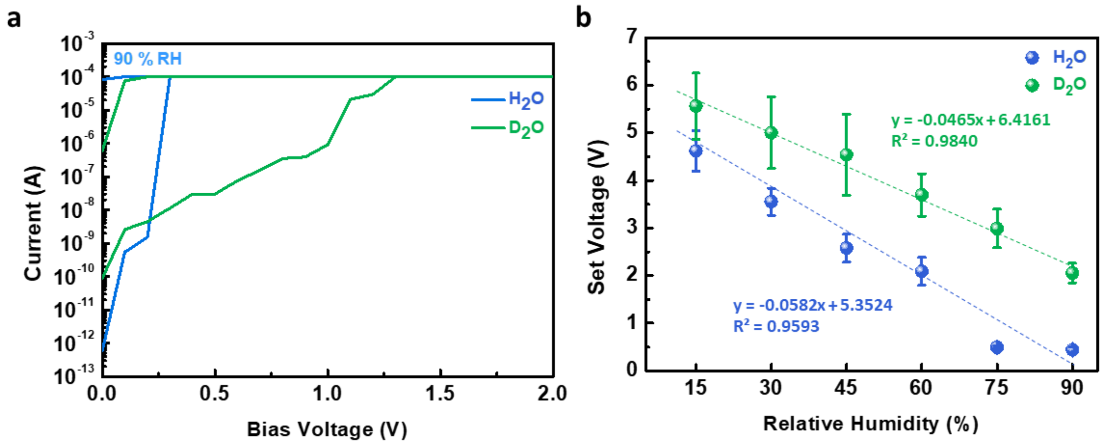

Supplementary Figure 9 | Comparison of the set voltages in H_2O and D_2O condition. a, I-V characteristics of the Y7C peptide memristor in H_2O (blue) and D_2O (green) condition at 90 % RH. **b,** The set voltages of the Y7C peptide memristor as a function of the RH of H_2O (blue) and D_2O (green). Dotted lines indicate linear fitting of the experimental results. The error bars represent mean ± SD.

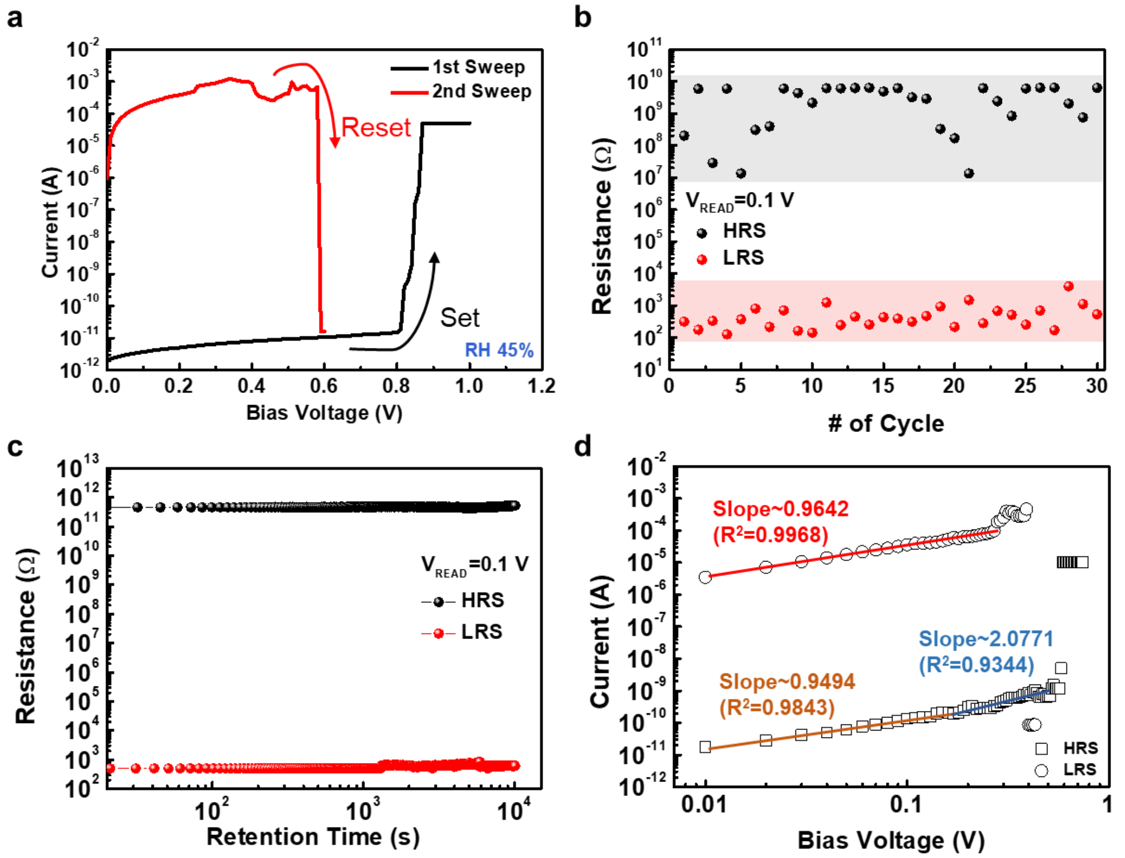

Supplementary Figure 10 | Unipolar resistive switching characteristics of the Y7C memristor. a, IV characteristics of the first and second sweeps at 45 % RH. The set and reset processes are indicated by black and red arrows, respectively. After the set process from the HRS to the LRS, the device underwent a reset process from the LRS to the HRS through a subsequent positive sweep. **b,** Switching endurance test up to 30 cycles. The regions of the HRS and the LRS are shown in black and red, respectively. The resistance of each state was measured at 0.1 V. **c,** Data retention test for 10^4 s. There is no degradation of data for both the LRS and the HRS, showing stable retention characteristics. **d,** Double-logarithmic fitting of the I–V characteristics. The HRS region, transition region and LRS region are fitted to brown, blue and red lines, respectively. The slopes and coefficients of determination for each fitting line are displayed in corresponding colors.

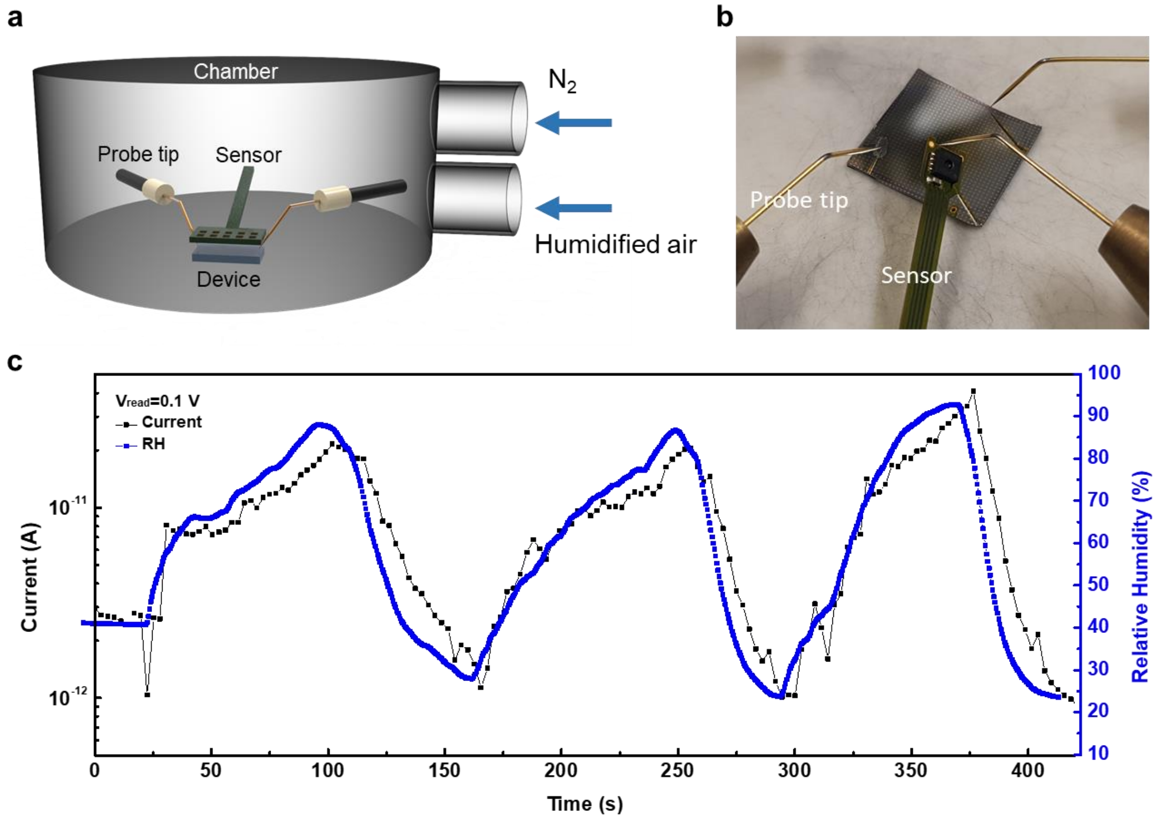

Supplementary Figure 11 | Dynamic measurement of the current change by RH control. **a,** Schematic of the probe chamber with the humidity sensor. N_2 and humidified air were injected to control the RH. **b,** Photograph of the probing system. The humidity sensor was located adjacent to the device for simultaneous detection. The distance from the sensor to the device is less than 0.5 cm. **c,** Humidity dependence of the transient current of the Y7C memristor. Dynamic measurements of the current and RH were carried out with frequencies of 0.4 and 2 Hz, respectively. The rate of the current change per relative humidity is 3.5 e^{-13} A/%. The current response to a change in RH is immediate and reversible.

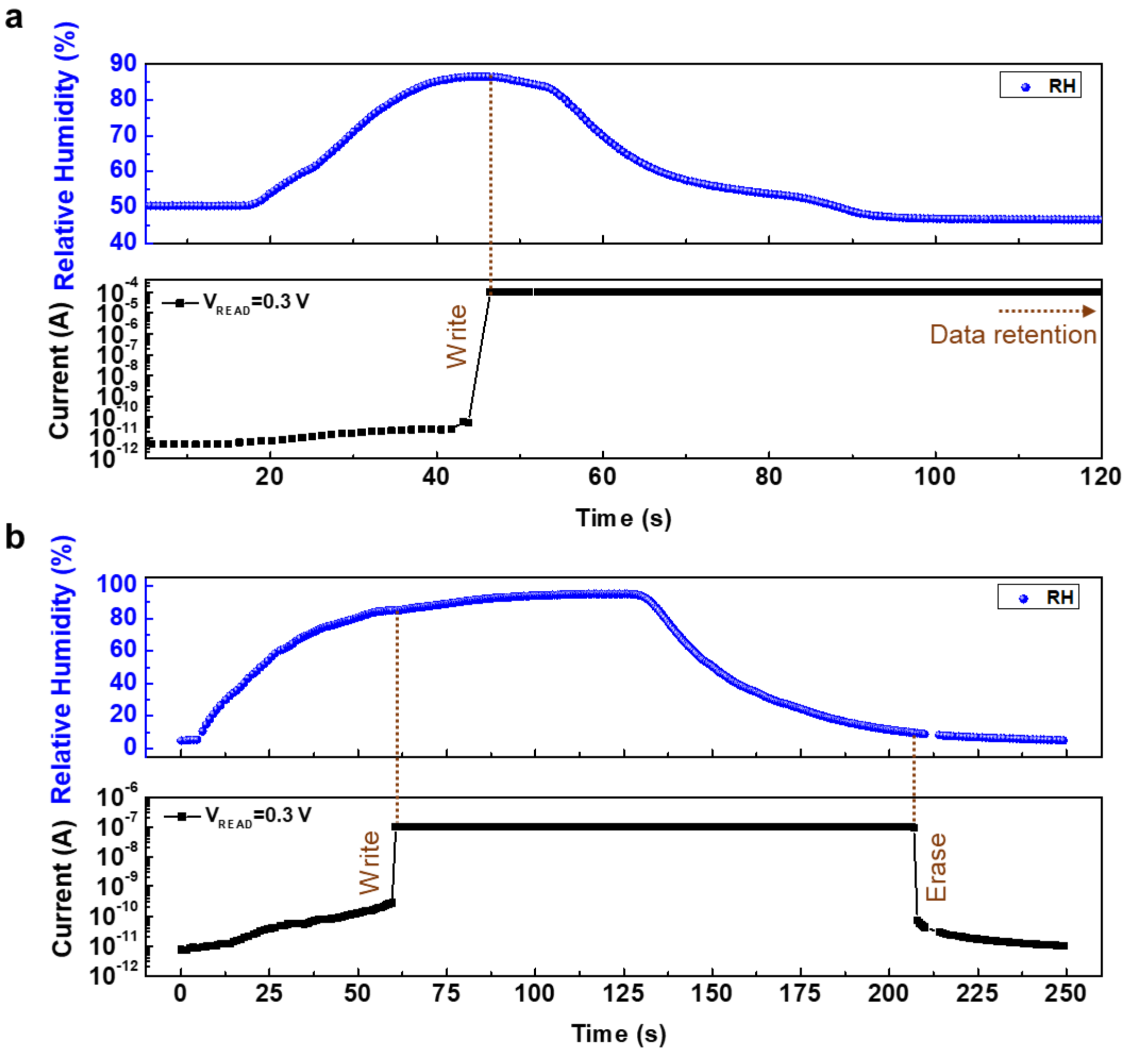

Supplementary Figure 12 | Resistive switching operation of the Y7C memristor controlled by the humidity input. a, Write operation and data retention. An RH pulse with an offset of 50 % was applied, analogous to a positive voltage sweep in bias mode. The write operation (set) occurred at 86 % RH, and the Y7C memristor maintained the LRS after the RH decreased to 50 %. **b,** Write and erase operation. An RH pulse with an offset of 5 % was applied. The write operation occurred at 86 % RH, and the erase operation (reset) occurred at 10 % RH. The points of the write and erase operations in the current graph are matched to the RH graph with a brown dotted line.

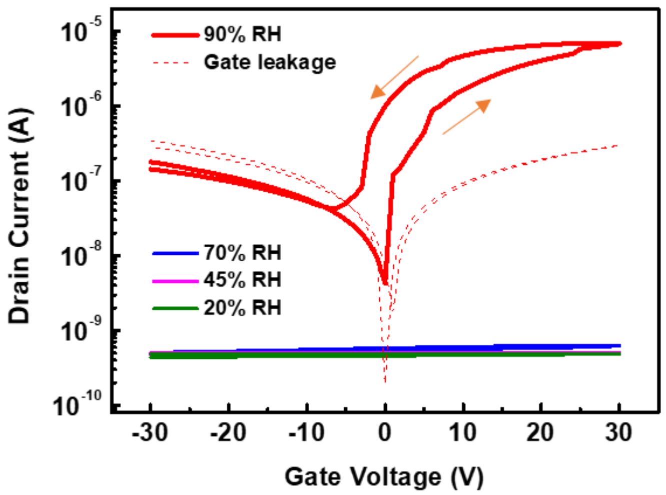

Supplementary Figure 13 | I_{ds}-V_{gs} characteristics of the proton-activated artificial synapse based on the Y7C peptide at various RHs. The transfer curve was observed only at 90 % RH due to the activated gating effect of the Y7C film. Despite the considerable level of the gate leakage, a current modulation of over 10^2 by the Y7C film occurred. The forward sweep and reverse sweep are indicated by arrows. In the cases of 70 %, 45 % and 20 % RH, no drain current modulation was observed. A drain voltage of 1 V was applied for all cases.

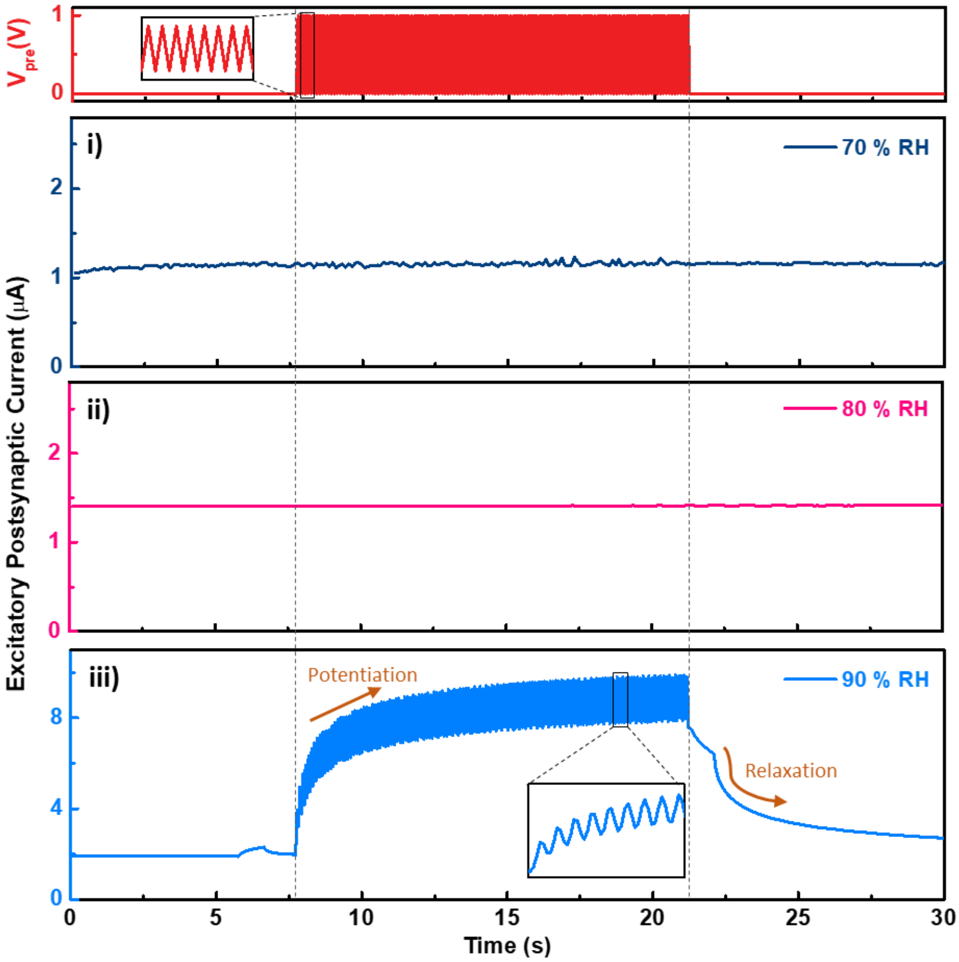

Supplementary Figure 14 | Proton-dependent plasticity of artificial synapses based on the Y7C peptide in fixed RH. The presynaptic voltage train (top) is applied and the following EPSCs are measured at fixed RHs of 70 % (i), 80 % (ii) and 90 % (iii).

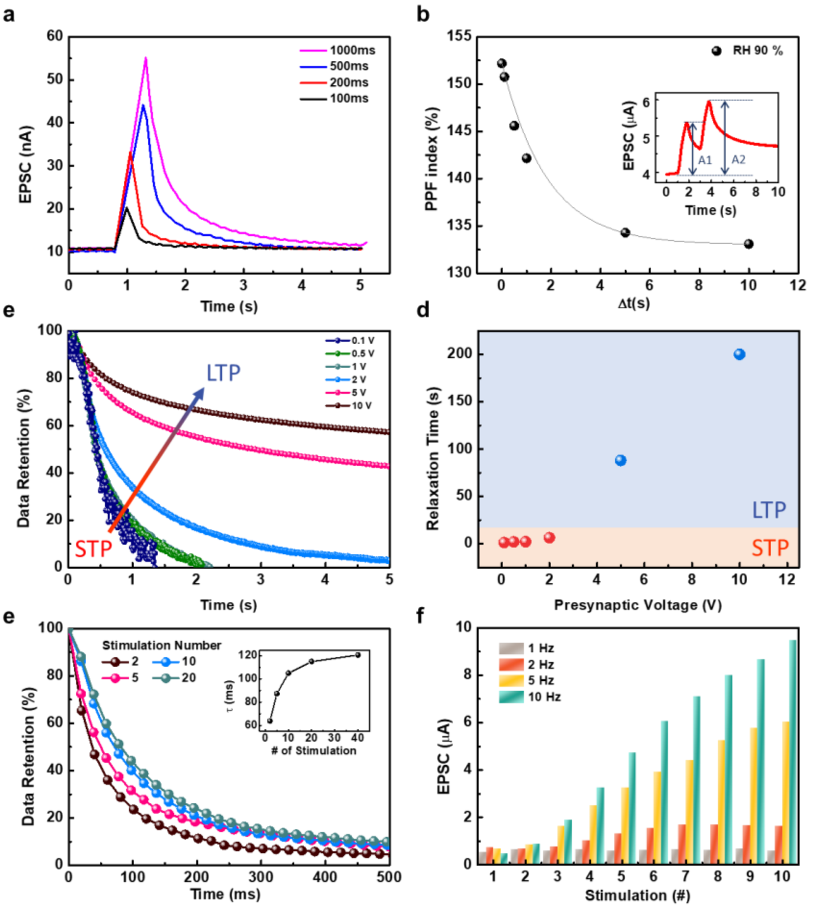

Supplementary Figure 15 | Synaptic plasticity of the proton-activated artificial synapse based on the Y7C peptide. a, Spike-duration-dependent plasticity. The amplitude of a presynaptic spike is 1 V. Increasing the duration of a presynaptic spike induced a higher peak value and longer decay time of the EPSC. **b,** Paired-pulse facilitation index, defined as A_2/A_1, as a function of the interval between two consecutive presynaptic spikes, Δt. The amplitude and duration of a presynaptic spike are 1 V and 1 s, respectively. The PPF index decreased when the spike interval was increased. **c,** Transition from short-term plasticity to long-term plasticity. Data retention after stimulated by presynaptic voltage pulse with various amplitude. Transition from shot-term plasticity (STP) to long-term plasticity (LTP) is indicated by an arrow. **d,** Relaxation time as a function of amplitude of presynaptic voltage. Relaxation time is defined as the time it takes to return to the pristine current after all data is lost. **e,** Normalized data retention curves with various numbers of presynaptic spikes. When the stimulation number increased, the decay time of the EPSC increased. Inset: relaxation time constant as a function of the stimulation number. **f,** The EPSC stimulated by 10 spikes with different pulse frequencies. The amplitude and duration of a presynaptic spike are 1 V and 100 ms, respectively. The modulation of the EPSC was considerably enhanced by stimulation with a higher frequency.

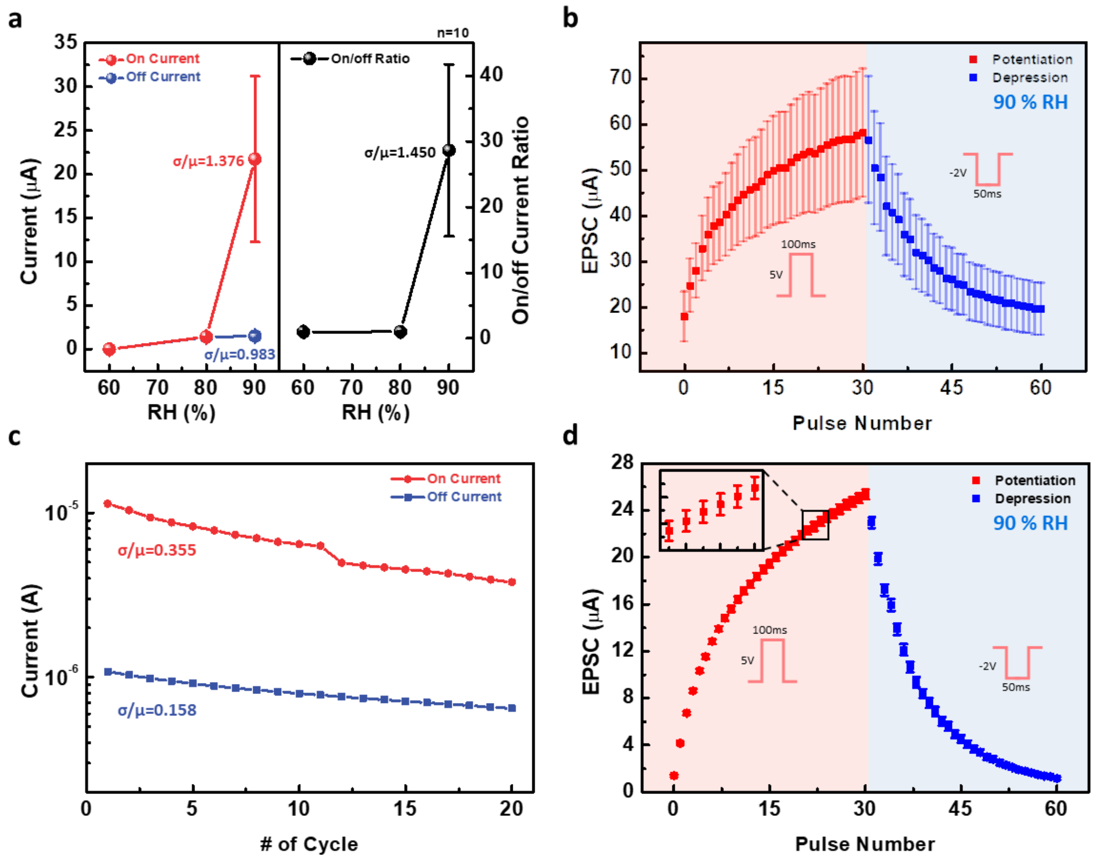

Supplementary Figure 16 | Spatial and temporal variation of the activated artificial synapse based on the Y7C peptide. a, On and off currents of transfer curves from 10 devices at gate voltages of 20 V and -5 V, respectively (left), and corresponding on/off current ratio (right). The spatial variations of the on current, off current and on/off ratio are 137.6 %, 98.3 % and 145 %, respectively. **b,** Potentiation and depression of EPSCs from 10 devices as 30 positive and negative pulses were applied. The spatial variation of the maximum conductance is 76.4 %. **c**, On and off currents of transfer curves from 20 cycles at gate voltages of 20 V and -5 V, respectively. The temporal variations of the on and off current are 35.5 % and 15.8 %, respectively. **d,** Potentiation and depression of the EPSCs from 10 cycles as 30 positive and negative pulses were applied. Inset is a magnified image of the potentiation curve. The error bars represent mean ± SE.

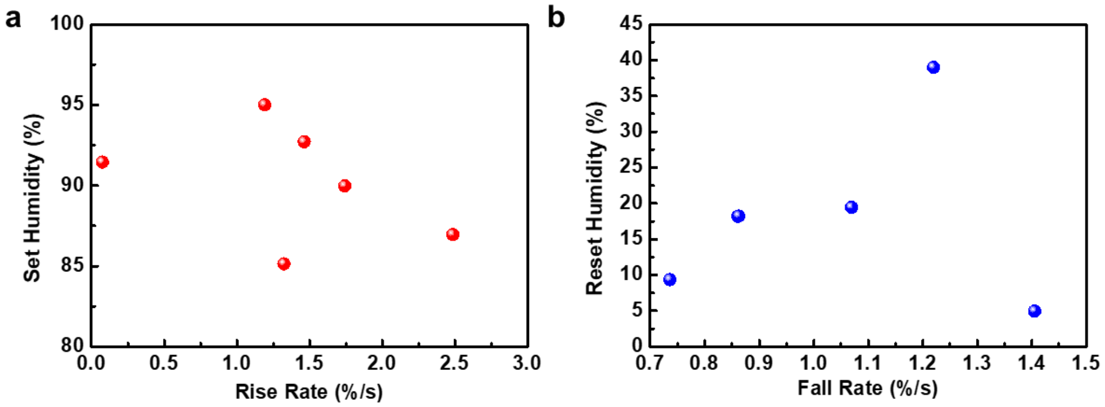

Supplementary Figure 17 | Set and reset humidity for the humidity mode of the Y7C memristor as a function of the RH scan rate. a, Set humidity as a function of the rise rate. **b,** Reset humidity as a function of the fall rate. The set and reset humidities do not show a dependence on the scan rate of the RH sweep.

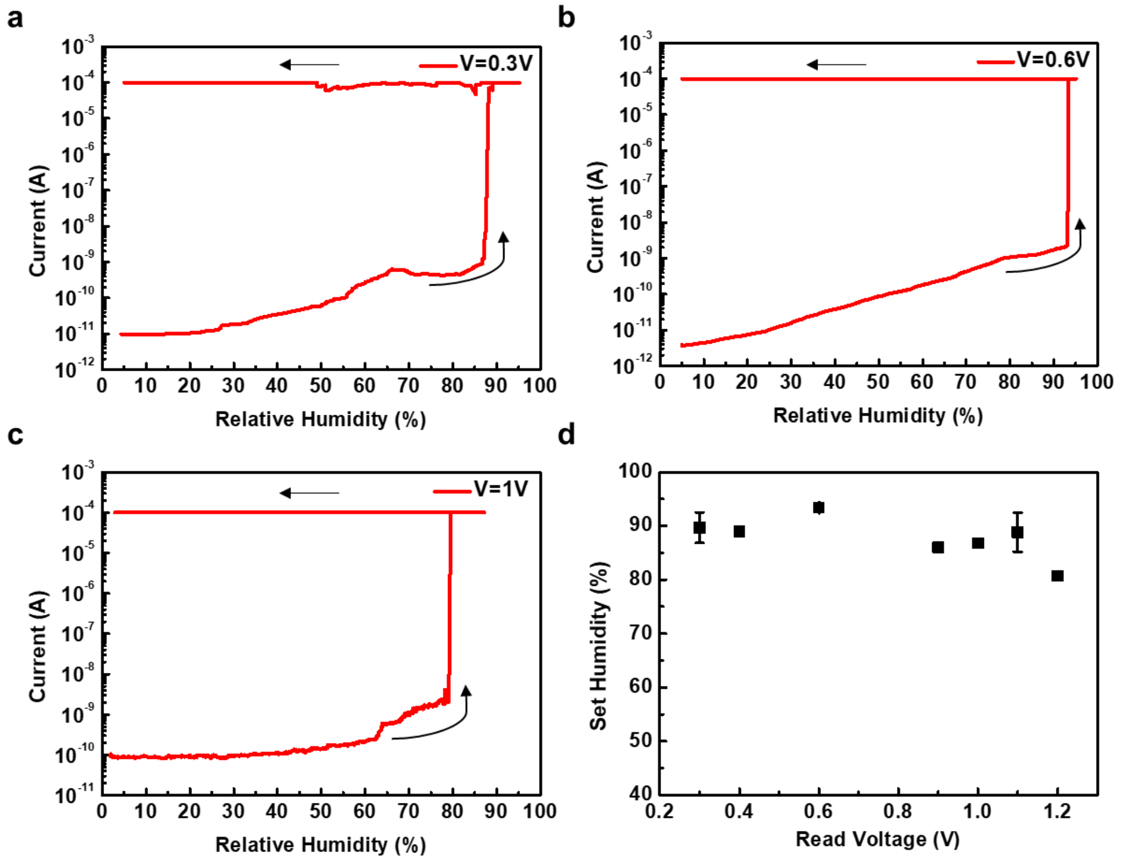

Supplementary Figure 18 | Set process for the humidity mode of the Y7C memristor with various read voltages. a-c, Resistive switching characteristics of the Y7C memristor with (**a**) V_{read} = 0.3 V, (**b**) V_{read} = 0.6 V and (**c**) V_{read} =1 V. The RH sweep directions are indicated by arrows. **d,** Set humidity as a function of the read voltage. The humidity does not show a dependence on the read voltage of the Y7C memristor. The error bars represent mean ± SD.

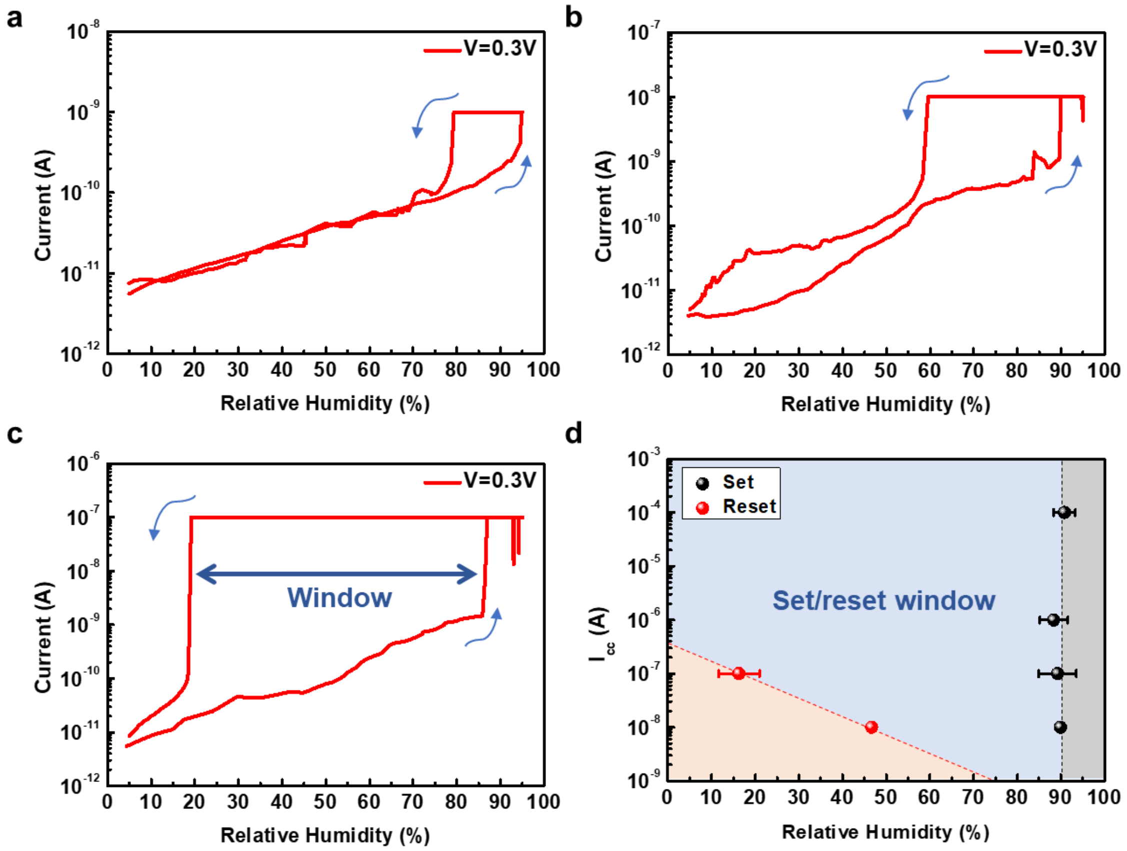

Supplementary Figure 19 | Set/reset window for the humidity mode of the Y7C memristor with various current compliance levels. a-c, Resistive switching characteristics of the Y7C memristor with (**a**) $I_{cc} = 10^{-9}$A, (**b**) $I_{cc} = 10^{-8}$A and (**c**) $I_{cc} = 10^{-7}$A. The RH sweep directions are indicated by blue arrows. **d,** Set/reset humidity plotted on the I_{cc}-RH graph. The region between the set and reset humidity is defined as the set/reset window indicated by a navy double-headed arrow in **c**. When the current compliance level decreased, the set/reset window decreased due to the increased reset humidity. The error bars represent the mean ± SD.

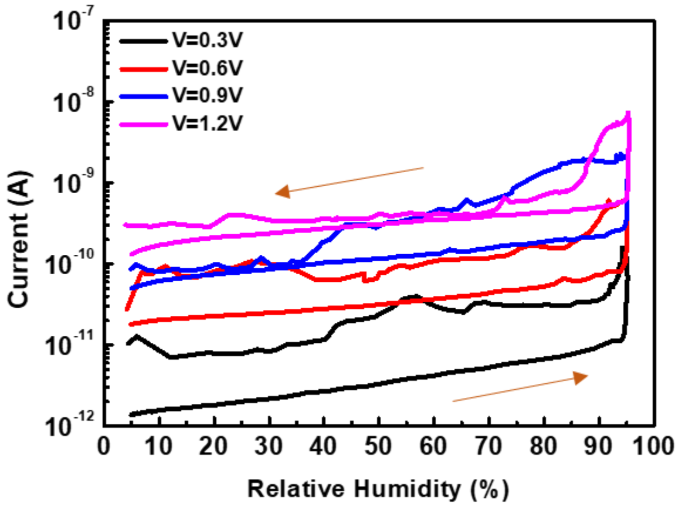

Supplementary Figure 20 | Resistive switching characteristics for the humidity mode of the Y7C memristor with the Au top electrode under various read voltage biases. The forward (5 %→95 %) and reverse (95 %→5 %) RH sweeps are indicated with arrows. An abrupt transition from the HRS to the LRS is not observed, but some hysteresis is observed.

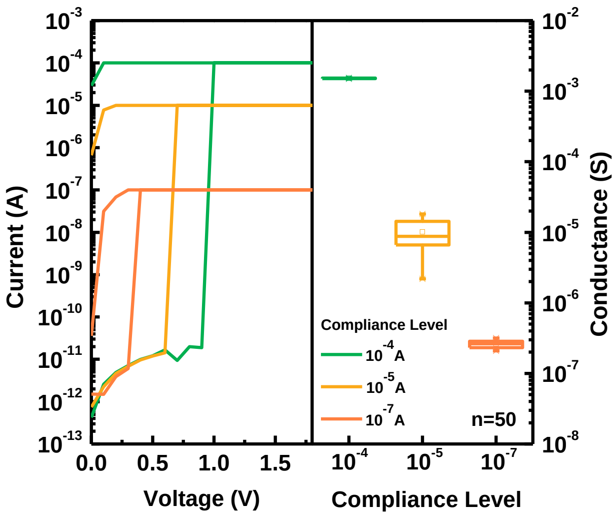

Supplementary Figure 21 | Conductance level adjusted by current compliance level during set process. IV characteristics of set process (left) and corresponding LRS conductance (right) were measured with different current compliance levels of 10^{-4} A (green), 10^{-5} A (yellow) and 10^{-7} A (orange).

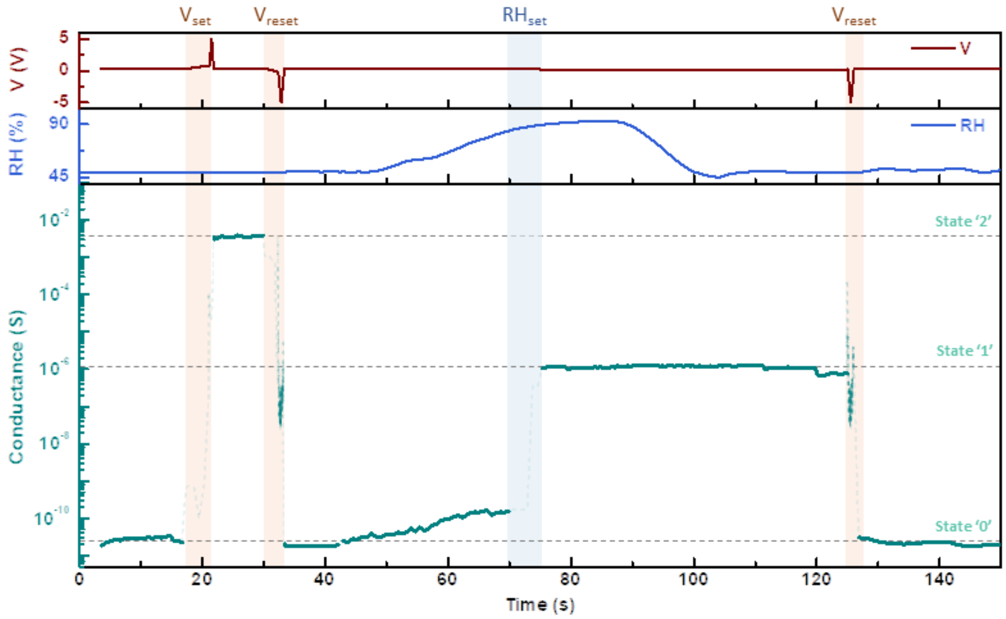

Supplementary Figure 22 | Bimodal operation of the Y7C peptide memristor in which bias mode and humidity mode are distinguished. Voltage pulse, RH pulse and corresponding current output are represented by brown, cyan and turquoise, respectively.

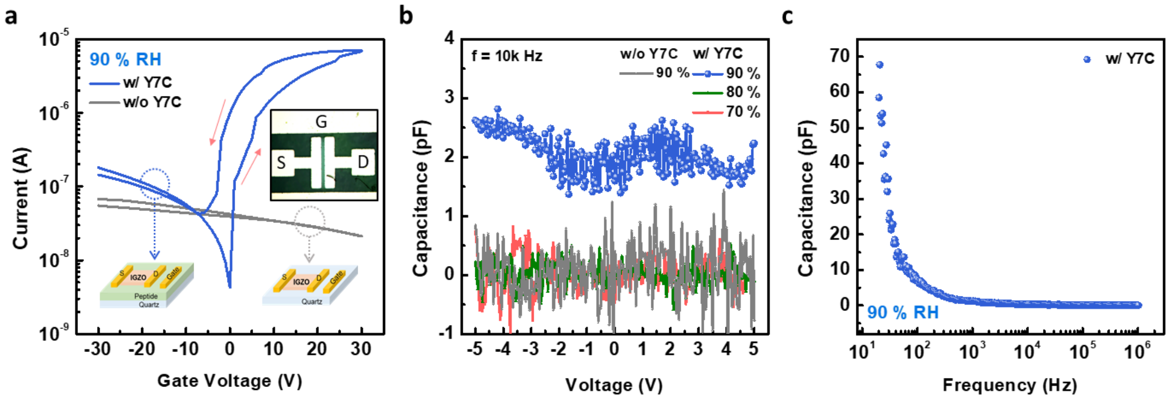

Supplementary Figure 23 | Proton activation and deactivation of electrostatic coupling effect. a, Transfer characteristics of the artificial synapses with (blue) and without (gray) the Y7C layer at 95 % RH. Drain voltage of 1 V was applied. Insets show an optical microscopy image (upper right) and device schematics of the devices with the Y7C layer (bottom left) and without the Y7C layer (bottom right). **b,** Capacitance–voltage characteristics of the artificial synapses with the Y7C layer at 70 % (red), 80 % (green) and 90 % (blue), and without the Y7C layer at 90 % RH (gray). The fluctuations of the alternating positive and negative values are noise. **c,** Capacitance-frequency characteristics of the artificial synapse with the Y7C layer at 95 % RH. Frequency sweep ranges from 20 Hz to 1 MHz.

Category	Material	Condition	Conductivity	Reference
Polymer	Nafion 117	30°C, soaked	77 mS/cm	(31)
	SPTES/SPBI	35°C, 85% RH	55 mS/cm	(32)
	SPBI	30°C, soaked	54 mS/cm	(33)
	SPEEK	20°C, soaked	45 mS/cm	(34)
Metal-Organic Framework	CPM-103a	22.5°C, 98% RH	58 mS/cm	(35)
	Fe-CAT-5	25°C, 98% RH	50 mS/cm	(36)
	UiO-66$(SO_3H)_2$	25°C, 90% RH	14 mS/cm	(37)
	$(NH_4)_2(adp)[Zn_2(ox)_3]3H_2O$	25°C, 98% RH	8 mS/cm	(38)
	Cd-5TIA	28°C, 98% RH	0.36 mS/cm	(39)
	FJU-31@Hg	30°C, 98% RH	0.05 mS/cm	(40)
Biomaterial	Shark jelly protein	RT, anhydrous	2 mS/cm	(41)
	Eumelanin	RT, 90% RH	0.71 mS/cm	(30)
	Reflectin protein	30°C, 90% RH	0.1 mS/cm	(6)
	Serum albumin	RT, soaked	0.05 mS/cm	(42)
**Our work**	**YYACAYY**	**22°C, 90% RH**	**17.6 mS/cm**	

Supplementary Table 1 | Comparison of the proton conductivities of representative proton-conducting materials. The Y7C peptide exhibited a considerably higher proton conductivity than reported metal-organic frameworks and biomaterials, and the proton conductivity was even comparable to the proton conductivities of polymers including Nafion, which is the state-of-the-art proton conductor.[6,30-42]

Supplementary References

1 Huggins, R. A. Simple method to determine electronic and ionic components of the conductivity in mixed conductors a review. *Ionics* **8**, 300-313 (2002).

2 Taylor, S. R. & Gileadi, E. Physical interpretation of the Warburg impedance. *Corrosion* **51**, 664-671 (1995).

3 Soboleva, T. Y. *et al.* Investigation of the through-plane impedance technique for evaluation of anisotropy of proton conducting polymer membranes. *J. Electroanal. Chem.* **622**, 145-152 (2008).

4 Lee, J. *et al.* Proton conduction in a tyrosine-rich peptide/manganese oxide hybrid nanofilm. *Adv. Funct. Mater.* **27**, 1702185 (2017).

5 Garrido, L., Lopez-Gonzalez, M., Tlenkopatchev, M. & Riande, E. Proton diffusion in polyelectrolytes based on hydrogenated polynorbornenes with imide side groups in the repeat unit as determined by NMR and impedance spectroscopies. *J. Membr. Sci.* **380**, 199-207 (2011).

6 Ordinario, D. D. *et al.* Bulk protonic conductivity in a cephalopod structural protein. *Nat. Chem.* **6**, 596-602 (2014).

7 Lundblad, R. L. *Chemical modification of biological polymers*. (CRC press, 2011).

8 Eckhardt, S. *et al.* Nanobio silver: its interactions with peptides and bacteria, and its uses in medicine. *Chem. Rev.* **113**, 4708-4754 (2013).

9 Kracht, S. *et al.* Electron transfer in peptides: on the formation of silver nanoparticles. *Angew. Chem. Int. Ed.* **54**, 2912-2916 (2015).

10 Henglein, A. Physicochemical properties of small metal particles in solution: "microelectrode" reactions chemisorption, composite metal particles, and the atom-to-metal transition. *J. Phys. Chem.* **97**, 5457-5471 (1993).

11 Yang, Y. C., Pan, F., Liu, Q., Liu, M. & Zeng, F. Fully room-temperature-fabricated nonvolatile resistive memory for ultrafast and high-density memory application. *Nano Lett.* **9**, 1636-1643 (2009).

12 Tseng, R. J. *et al.* Digital memory device based on tobacco mosaic virus conjugated with nanoparticles. *Nat. Nanotechnol.* **1**, 72-77 (2006).

13 Jo, S. H. *et al.* Nanoscale memristor device as synapse in neuromorphic systems. *Nano Lett.* **10**, 1297-1301 (2010).

14 Choi, S. *et al.* SiGe epitaxial memory for neuromorphic computing with reproducible high performance based on engineered dislocations. *Nat. Mater.* **17**, 335-340 (2018).

15 Fuller, E. J. *et al.* Parallel programming of an ionic floating-gate memory array for scalable neuromorphic computing. *Science* **364**, 570-574 (2019).

16 Waser, R., Dittmann, R., Staikov, G. & Szot, K. Redox-based resistive switching memories–nanoionic mechanisms, prospects, and challenges. *Adv. Mater.* **21**, 2632-2663 (2009).

17 Meyer, T. J., Huynh, M. H. V. & Thorp, H. H. The possible role of proton-coupled electron transfer (PCET) in water oxidation by photosystem II. *Angew. Chem. Int. Ed.* **46**, 5284-5304 (2007).

18 Jang, H. S. *et al.* Tyrosine-mediated two-dimensional peptide assembly and its role as a bio-inspired catalytic scaffold. *Nat. Commun.* **5**, 1-11 (2014).

19 Sjödin, M. *et al.* Switching the redox mechanism: models for proton-coupled electron transfer from tyrosine and tryptophan. *J. Am. Chem. Soc.* **127**, 3855-3863 (2005).

20 Ju, M. *et al.* Quantitative analysis of the coupling between proton and electron transport in peptide/manganese oxide hybrid films. *Phys. Chem. Chem. Phys.* **22**, 7537-7545 (2020).

21 Xie, J., Lee, J. Y., Wang, D. I. & Ting, Y. P. Silver nanoplates: from biological to biomimetic synthesis. *ACS Nano* **1**, 429-439 (2007).

22 Zhang, C. C. *et al.* Convertible resistive switching characteristics between memory switching and threshold switching in a single ferritin-based memristor. *Chem. Commun.* **52**, 4828-4831 (2016).

23 Porro, S. *et al.* A multi-level memristor based on atomic layer deposition of iron oxide. *Nanotechnology* **29**, 495201 (2018).

24 Wang, Z. *et al.* Threshold switching of Ag or Cu in dielectrics: materials, mechanism, and applications. *Adv. Funct. Mater.* **28**, 1704862 (2018).

25 Kwon, J. Y. & Jeong, J. K. Recent progress in high performance and reliable n-type transition metal oxide-based thin film transistors. *Semicond. Sci. Technol.* **30**, 024002 (2015).

26 Zhou, J., Liu, N., Zhu, L., Shi, Y. & Wan, Q. Energy-efficient artificial synapses based on flexible IGZO electric-double-layer transistors. *IEEE Electron Device Lett.* **36**, 198-200 (2014).

27 Sun, J. *et al.* Optoelectronic synapse based on IGZO-alkylated graphene oxide hybrid structure. *Adv. Funct. Mater.* **28**, 1804397 (2018).

28 Ohno, T. *et al.* Short-term plasticity and long-term potentiation mimicked in single inorganic synapses. *Nat. Mater.* **10**, 591-595 (2011).

29 Sung, T. *et al.* Effects of proton conduction on dielectric properties of peptides. *RSC Adv.* **8**, 34047-34055 (2018).

30 Wünsche, J. *et al.* Protonic and electronic transport in hydrated thin films of the pigment eumelanin. *Chem. Mater.* **27**, 436-442 (2015).

31 Wang, C. *et al.* Fluorene-based poly(arylene ether sulfone)s containing clustered flexible pendant sulfonic acids as proton exchange membranes. *Macromolecules* **44**, 7296-7306 (2011).

32 Bai, Z. *et al.* Proton exchange membranes based on sulfonated polyarylenethioethersulfone and sulfonated polybenzimidazole for fuel cell applications. *J. Membr. Sci.* **305**, 69-76 (2007).

33 Glipa, X., El Haddad, M., Jones, D. J. & Rozière, J. Synthesis and characterisation of sulfonated polybenzimidazole: a highly conducting proton exchange polymer. *Solid State Ion.* **97**, 323-331 (1997).

34 Bauer, B. *et al.* Electrochemical characterisation of sulfonated polyetherketone membranes. *J. New Mat. Electr. Sys.* **3**, 93-98 (2000).

35 Zhai, Q. G. *et al.* Cooperative crystallization of heterometallic indium–chromium metal–organic polyhedra and their fast proton conductivity. *Angew. Chem. Int. Ed.* **54**, 7886-7890 (2015).

36 Nguyen, N. T. T. *et al.* Three-dimensional metal-catecholate frameworks and their ultrahigh proton conductivity. *J. Am. Chem. Soc.* **137**, 15394-15397 (2015).

37 Phang, W. J. *et al.* Superprotonic conductivity of a UiO-66 framework functionalized with sulfonic acid groups by facile postsynthetic oxidation. *Angew. Chem. Int. Ed.* **54**, 5142-5146 (2015).

38 Sadakiyo, M., Yamada, T. & Kitagawa, H. Rational designs for highly proton-conductive metal-organic frameworks. *J. Am. Chem. Soc.* **131**, 9906-9907 (2009).

39 Panda, T., Kundu, T. & Banerjee, R. Self-assembled one dimensional functionalized metal-organic nanotubes (MONTs) for proton conduction. *Chem. Commun.* **48**, 5464-5466 (2012).

40 Ye, Y. *et al.* Metal–organic frameworks with a large breathing effect to host hydroxyl compounds for high anhydrous proton conductivity over a wide temperature range from subzero to 125° C. *J. Mater. Chem. A* **4**, 4062-4070 (2016).

41 Josberger, E. E. *et al.* Proton conductivity in ampullae of Lorenzini jelly. *Sci. Adv.* **2**, e1600112 (2016).

42 Amdursky, N., Wang, X., Meredith, P., Bradley, D. D. & Stevens, M. M. Long-range proton conduction across free-standing serum albumin mats. *Adv. Mater.* **28**, 2692-2698 (2016).
